# ﻿Revised checklist of endemic vascular plants of Kazakhstan

**DOI:** 10.3897/phytokeys.238.114475

**Published:** 2024-02-28

**Authors:** Serik A. Kubentayev, Daniyar T. Alibekov, Yuri V. Perezhogin, Georgy A. Lazkov, Andrey N. Kupriyanov, Alexander L. Ebel, Klara S. Izbastina, Olga V. Borodulina, Balsulu B. Kubentayeva

**Affiliations:** 1 Astana Botanical Garden, 16 Orynbor Str., 010016, Astana, Kazakhstan Astana Botanical Garden Astana Kazakhstan; 2 Kostanay Regional University named after A. Baitursynova, 47 Baytursynov Str., 110000, Kostanay, Kazakhstan Kostanay Regional University named after A. Baitursynova Kostanay Kazakhstan; 3 Institute of Biology of the National Academy of Sciences of the Republic of Kyrgyzstan, Bishkek 720011, Kyrgyzstan Institute of Biology of the National Academy of Sciences of the Republic of Kyrgyzstan Bishkek Kyrgyzstan; 4 Research Centre for Ecology and Environment of Central Asia, Bishkek 720040, Kyrgyzstan Research Centre for Ecology and Environment of Central Asia Bishkek Kyrgyzstan; 5 Federal Research Center of Coal and Coal Chemistry of Siberian Branch of the Russian Academy of Sciences, 18 Sovetsky Ave., 650000, Kemerovo, Russia Federal Research Center of Coal and Coal Chemistry of Siberian Branch of the Russian Academy of Sciences Kemerovo Russia; 6 Tomsk State University, Lenin Ave. 36, 634050 Tomsk, Russia Tomsk State University Tomsk Russia; 7 Central Siberian Botanical Garden, Siberian Branch of the Russian Academy of Sciences, Zolotodolinskaya Str. 101, 630090 Novosibirsk, Russia Central Siberian Botanical Garden, Siberian Branch of the Russian Academy of Sciences Novosibirsk Russia; 8 S. Seifullin Kazakh Agrotechnical Research University, 62 Zhengis Ave, 010000, Astana, Kazakhstan S. Seifullin Kazakh Agrotechnical Research University Astana Kazakhstan

**Keywords:** Biodiversity hotspots, Central Asia, conservation, endemism, floristic division

## Abstract

We compiled a checklist of endemic vascular plants occurring in Kazakhstan, employing an exhaustive examination of literature sources, herbarium collections, databases and field observations. Our study reveals that 451 taxa can be considered endemic to Kazakhstan, constituting 7.97% of the total vascular plant diversity in the country. These endemic taxa, originating from 139 genera and 34 families, predominantly thrive in the southern regions of Kazakhstan, specifically in the mountain ridges of the Kazakh part of the Tian Shan, including Karatau (123 taxa), Dzungarian Alatau (80 taxa) and Trans-Ili and Kungey Alatau (50 taxa). Notably, 107 endemic species are granted legal protection. Detailed information regarding life form, life cycle, conservation status and geographical distribution across floristic regions was meticulously compiled for each endemic taxon. Of the six groups of life forms, herbs include the highest part of endemic taxa (367 taxa), followed by dwarf semishrubs (25 taxa), shrubs (23 taxa), subshrubs (20 taxa), undershrubs (13 taxa) and trees (3 taxa). The observed life cycles are perennials (408 taxa), annuals (33 taxa) and biennials (10 taxa). This paper serves as a fundamental groundwork for prospective investigations aimed at assessing population sizes and hotspots of plant endemism throughout Kazakhstan, crucial for determining conservation status of endemic plants.

## ﻿Introduction

Endemic plants hold particular importance in studying the history of flora and vegetation of diverse geographical regions, since they represent an important element of biodiversity and serve as vital benchmarks for identifying areas of high biodiversity value. In recent years, much attention has been paid to the study of endemic plants, as evidenced by a large number of scientific publications (Tojibaev et al. 2020a; Baasanmunkh et al. 2022; Erst et al. 2022; Chung et al. 2023; Villaseñor et al. 2023).

Kazakhstan occupies a central position within Eurasia and holds a notable distinction of being the ninth largest country globally, with 2,724,900 km^2^ of land area. The territory of Kazakhstan is characterised by a remarkable ecological heterogeneity (Abdulina 1999; Akzhigitova et al. 2003), marked by prominent zonal boundaries, notably the demarcation between the cold-temperate and temperate regions of Northern Eurasia and the Irano-Turanian warm region with the Mediterranean-like type of climate, the latter encompassing the southern part of Kazakhstan.

The remarkable diversity of natural conditions in Kazakhstan contributes to the exceptional richness of its flora, its notable originality and a significant number of endemic plant species in Kazakhstan. According to the latest inventory, 5,658 vascular plant species, representing 159 families and 1,067 genera, occur in the country (Abdulina 1999).

The investigation of endemic plant species, which represent a vital and highly vulnerable component of biodiversity, has garnered significant attention in numerous countries. The number of endemic plants in the countries neighbouring Kazakhstan varies, with China exhibiting the highest number of endemic species at 14,939 (Huang et al. 2011). There are over 2,700 endemic taxa in Russia (Kamelin and Budantsev 2019), Mongolia has 102 taxa (Baasanmunkh et al. 2022), Kyrgyzstan has 393 taxa (Lazkov and Sultanova 2014) and Uzbekistan has 378 taxa (Sennikov et al. 2016). Based on a comprehensive review of the “Flora of Kazakhstan” (Pavlov 1956–1966), Goloskokov (1969) counted 760 endemic species from 199 genera and 47 families in Kazakhstan. Otherwise, various sources estimated the presence of 709 to 823 species of endemic plants in Kazakhstan (Pavlov 1956–1966; Bykov 1966; Goloskokov 1969; Baitenov 2001; Gemedjieva et al. 2010).

The scientific literature contains a substantial body of work focused on the investigation of endemic taxa in Kazakhstan. However, these publications predominantly revolve around limited geographical areas, such as specific mountain ranges, floristic regions or administrative divisions (Pavlov 1970; Goloskokov 1979; Baitenov 1982; Anapiev 1996; Bakeev and Atikeeva 2015; Ishmuratova et al. 2015, 2016a, 2016b; Sadyrova et al. 2017; Mukhtubaeva et al. 2017; Kupriyanov 2022). Reports focusing on endemic plant species within certain families and genera have also been published, including studies on Poaceae (Kupriyanov et al. 2018), Apiaceae (Klyuykov and Ukrainskaya 2018), Asteraceae (Kupriyanov 2018), Ranunculaceae (Shchegoleva 2019a), Chenopodiaceae (Osmanali et al. 2019) and *Oxytropis* (Perezhogin et al. 2020).

The available information regarding the composition of endemic plant species in Kazakhstan, as documented in the “Flora of Kazakhstan” (Pavlov 1956–1966) and other related sources (Bykov 1966; Goloskokov 1969), is largely outdated. Since then, numerous species previously classified as endemic have been discovered beyond the borders of Kazakhstan or reduced to synonyms. In addition, in the last 10 years alone, more than 25 species of endemic plants have been described as new to science from the territory of Kazakhstan, for example: six species of *Tulipa* (*T.annae* J.de Groot & Zonn, *T.auliekolica* Perezhogin, *T.dianaeverettiae* J.de Groot & Zonn., *T.turgaica* Perezhogin, *T.salsola* Rukšāns & Zubov, *T.ivasczenkoae* Epiktetov & Belyalov) (Epiktetov and Belyalov 2013; Perezhogin 2013; de Groot and Zonneveld 2020; Rukšāns and Zubov 2022); five apomictic species of *Taraxacum* (*T.atrochlorinum* Kirschner & Štěpánek, *T.corvinum* Kirschner & Štěpánek, *T.dzhungaricola* Kirschner & Štěpánek, *T.sublilacinum* Kirschner & Štěpánek) (Kirschner and Štěpánek 2017); three species of *Allium: A. koksuense* R.M.Fritsch, N.Friesen & S.V.Smirn., *A.lepsicum* R.M.Fritsch, N.Friesen & S.V.Smirn. and *A.toksanbaicum* N.Friesen & Veselova (Friesen et al. 2021a, 2021b); two species of *Hedysarum* (*H.tarbagataicum* Knjaz. and *H.ulutavicum* Knjaz.) (Knyazev 2019); *Myosotiskazakhstanica* O.D.Nikif. (Nikiforova 2018); *Gageaalmaatensis* Levichev, A.Peterson & J.Peterson (Peterson et al. 2016); *Galatellabectauatensis* Kupr. & Koroljuk (Kupriyanov and Korolyuk 2013); *Rhaponticoideszaissanica* Kupr., A.L.Ebel & Khrustaleva (Kupriyanov 2020); *Astragalussaphronovae* Kulikov (Kulikov 2014); *Phlomismindshelkensis* Lazkov (Lazkov 2014); *Phlomoidesboroldaica* A.L.Ebel (Ebel et al. 2019); *Fritillariakolbintsevii* Rukšāns & Zubov (Rukšāns and Zubov 2021; *Galiumzaisanicum* Pinzhenina & Kupr. (Pinzhenina and Kupriyanov 2023); *Prangosmulticostata* Kljuykov & Lyskov (Lyskov et al. 2016), *Sphaenolobiumkorovinii* Pimenov & Kljuykov (Pimenov and Kljuykov 2014) and *Nitrariailiensis* Banaev & Tomoshevich (Banaev et al. 2023).

Consequently, the current knowledge regarding the species diversity of endemic plants in Kazakhstan remains poorly available. In order to address this knowledge gap, our research endeavour aimed to compile the checklist of endemic vascular plants in Kazakhstan, based on an extensive analysis of literary sources, comprehensive revision of herbarium collections and data from field observations.

## ﻿Materials and methods

For the compilation of an endemic plant checklist in Kazakhstan, extensive literature sources were consulted. Initially, nine volumes of the “Flora of Kazakhstan” (Pavlov 1956, 1958, 1960, 1961a, 1961b, 1963, 1964, 1965, 1966) were utilised, alongside the complete list of the country’s flora (Abdulina 1999). The broad-scale inventory of Central Asian plants, “Conspectus Florae Asiae Mediae”, spanning 11 volumes (Kovalevskaya 1968, 1971; Bondarenko and Nabiev 1972; Pakhomova 1974, 1976; Kamelin et al. 1981; Adylov 1983, 1987; Nabiev 1986; Adylov and Zuckerwanik 1993; Khassanov 2015), was also referenced. Additionally, the “Plants of Central Asia” series, consisting of 16 volumes (Grubov 1963–2008), was incorporated. Reports detailing endemic plants within specific geographical and administrative regions of Kazakhstan were used (Pavlov 1970; Goloskokov 1979; Baitenov 1982; Anapiev 1996; Bakeev and Atikeeva 2015; Ishmuratova et al. 2015, 2016a, 2016b; Mukhtubaeva et al. 2017; Sadyrova et al. 2017; Kupriyanov 2022). Furthermore, lists highlighting endemic plants within particular species-rich families and genera were considered, such as Ranunculaceae (Shchegoleva 2019a), Apiaceae (Klyuykov and Ukrainskaya 2018), Asteraceae (Kupriyanov 2018), *Achillea* (Kupriyanov and Kulemin 2023), *Oxytropis* (Perezhogin et al. 2020) and Chenopodiaceae (Suchorukow 2007; Osmanali et al. 2019). Lists encompassing endemic plants within broader geographical regions, which include parts of Kazakhstan, were also reviewed (Tolmachev 1974; Pyak et al. 2008; Tojibaev et al. 2020a; Erst et al. 2022). Additionally, we paid attention to the species described from Kazakhstan and new combinations published from 2013 to 2023, subsequent to the publication of the latest flora list by Abdulina (1999).

Following the compilation of a list of endemic taxa, we conducted a comprehensive re-assessment of the distribution of each species by cross-referencing published floristic records encompassing the administrative and geographical regions of Kazakhstan (Goloskokov 1949; Stepanova 1962; Karmysheva 1973, 1982; Pavlov 1980; Baitenov 1985; Pugachev 1994; Safronova 1996; Kotukhov 2005; Aralbay et al. 2006; Kadenova et al. 2008; Aipeisova 2012, 2013; Ishmuratova et al. 2016a; Kokoreva et al. 2018; Ivashchenko 2020; Kupriyanov 2020; Sitpayeva et al. 2020; Kubentayev et al. 2021; Orazov et al. 2022, 2024; Khasanov et al. 2023; Kulymbet et al. 2023; Osmonali et al. 2023; Sumbembayev et al. 2023). Furthermore, in order to clarify the presence of presumably endemic plants of Kazakhstan in neighbouring countries, we consulted floristic records of those territories (Kamelin 1990; Yakovlev 2003; Kulikov 2005; Wu et al. 2008; Ryabinina and Knyazev 2009; Lazkov and Sultanova 2014; Knyazev 2016; Nowak et al. 2020; Vaganov and Shmakov 2020; Sennikov and Tojibaev 2021; Baasanmunkh et al. 2022), as well as publications documenting the discovery of former Kazakhstan endemics outside their native range (Ho and Fu 1993; Yakovlev 2003; Kurtto et al. 2004; German 2005; Mavrodiev et al. 2005; German 2006a, 2006b; German et al. 2006; Belkin 2009; Sennikov et al. 2011; Soskov 2011; German et al. 2012; German et al. 2013; Byalt and Bubyreva 2014; German 2014; Nobis et al. 2014; Pimenov and Kljuykov 2014; Vesselova 2016; German and Al-Shehbaz 2017; Lazkov and Sennikov 2017a, 2017b; Nobis et al. 2017; Pimenov 2017; Golovanov et al. 2018; Golovanov and Knyazev 2019; Ma and Xu 2019; Shchegoleva et al. 2019b; Zolotukhin and Chkalov 2019; Ovchinnikova 2021; Tojibaev et al. 2022; Sennikov and Lazkov 2023; Vaganov 2023; Juramurodov et al. 2024).

To verify endemic taxa distributions, we employed systematic reports detailing the flora of Kazakhstan and its neighbouring regions (Baitenov 1977; German and Chen 2009; Kljuykov et al. 2018; Smirnov et al. 2018; Nobis et al. 2020; Pimenov 2020; German and Veselova 2022; Sennikov et al. 2023). Additionally, we conducted a thorough examination of specimens housed in various herbaria, including LE, MW, TK, TASH, MHA, SVER, KUZ, ALTB, NS, NSK and MOSP (herbarium acronyms according to Thiers (2023)), as well as the data sourced from the Global Biodiversity Information Facility (GBIF 2023), the International Legume Database and Information Service (ILDIS) (Roskov et al. 2009), BrassiBase: Introdcution to a novel database on Brassicaceae evolution. Plant & Cell Physiology (Kiefer et al. 2014), World Plants. Synonymic Checklist and Distribution of the World Flora (Hassler 1994–2024) and the Compositae Working Group (CWG) (2023).

Within the scope of this investigation, we provide a list and an analysis of national endemic vascular plants growing strictly within Kazakhstan (see Appendix 1). This study considers two taxonomic levels of endemic plants: species and subspecies; taxa with a rank lower than subspecies were not considered. Additionally, we present a separate list encompassing sub-endemic taxa (see Suppl. material 1). In this paper, sub-endemics refer to taxa that were formerly considered endemics, but subsequently found in a neighbouring country or countries, based on published literature or herbarium material. In addition, we present a list of former endemics of Kazakhstan reclassified as synonyms of taxa with broader geographical distributions (see Suppl. material 2).

**Figure 1.**
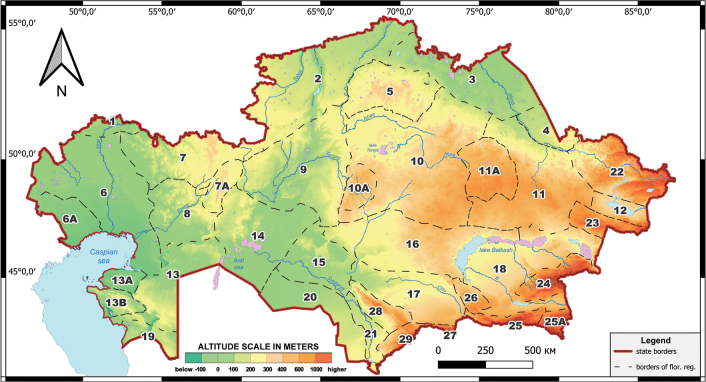
Map of the floristic division of Kazakhstan (Pavlov 1956): 1 – Syrt, 2 – Tobol-Ishim, 3 – Irtysh, 4 – Semipalatinsk pine forest, 5 – Kokchetav, 6 – Caspian Region, 6a – Bukeev, 7 – Aktobe, 7a – Mugojary, 8 – Emba, 9 – Turgay, 10 – Western Upland, 10a – Ulutau, 11 – Eastern Upland, 11a – Karkaraly, 12 – Zaysan, 13 – Northern Ustyrt, 13a – Buzachi, 13b – Mangyshlak, 14 – Aral Region, 15 – Kyzylorda, 16 – Betpak-Dala, 17 – Moiynkum, 18 – Balkhash-Alakol, 19 – Southern Ustyrt, 20 – Kyzylkum, 21 – Turkestan, 22 – Altai, 23 – Tarbagatai, 24 – Dzungarian Alatau, 25 – Trans-Ili Kungey Alatau, 25a – Ketmen-Terskey Alatau, 26 – Chu-Ili Range, 27 – Kyrgyz Alatau, 28 – Karatau, 29 – Western Tian Shan.

The distribution of each endemic taxon in Kazakhstan is given according to the floristic division of the country (Pavlov 1956). This division partitions Kazakhstan’s territory into 29 distinct floristic regions and seven subregions (Fig. 1).

The systematic order and taxonomic position of the families are based on the classification of angiosperms by APG IV (2016). The names of the accepted genera and species are mostly in accordance with Plants of the World Online (POWO 2023), with corrections according to recently-published taxonomic revisions. The authorship of species, genera and families has been critically cross-checked against the information provided in the International Plant Names Index (IPNI 2023).

## ﻿Results

Based on a rigorous revision of endemic vascular plants in Kazakhstan, a total of 451 taxa have been identified as endemic to the country (Appendix 1), which account for 7.97% of the total number (5,658 species) of vascular plants in Kazakhstan (Abdulina 1999). The endemic taxa recognised in this study belong to 139 genera and 34 families. Notably, Kazakhstan is home to five monotypic endemic genera, i.e. *Karatavia* Pimenov & Lavrova, *Botschantzevia* Nabiev, *Tschulaktavia* Bajtenov ex Pimenov & Kljuykov, *Cancriniella* Tzvelev and *Sauria* Bajtenov. No endemic families are present in the country.

**Figure 2.**
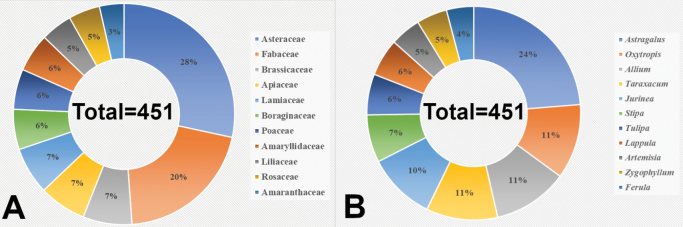
The largest families by the number of endemic taxa (**A**). The largest genera by the number of endemic taxa (**B**).

The greatest number of endemic taxa is registered in the following families: Asteraceae Bercht. & J.Presl (111 taxa from 29 genera), Fabaceae Lindl. (81 taxa from 6 genera), Apiaceae Lindl. (27 taxa from 13 genera), Lamiaceae Martinov (27 taxa from 8 genera), Boraginaceae Juss. (23 taxa from 10 genera), Brassicaceae Burnett (23 taxa from 8 genera), Amaryllidaceae J.St.-Hil. (22 taxa from 1 genus), Liliaceae Juss. (18 taxa from 3 genera), Rosaceae Juss. (17 taxa from 7 genera), Poaceae Barnhart (15 taxa from 8 genera) and Amaranthaceae Juss. (14 taxa from 10 genera) (Fig. 2A). The remaining 23 families are represented by one to 10 species each.

Genera with the greatest number of endemic taxa are as follows: *Astragalus* L. with 46 taxa, *Oxytropis* DC. with 22 taxa, *Allium* L. with 21 taxa, *Taraxacum* F.H.Wigg. with 20 taxa, *Jurinea* Cass. with 20 taxa, *Tulipa* L. with 13 taxa, *Lappula* Moench with 11 taxa, *Artemisia* L. with nine taxa, *Zygophyllum* L. with nine taxa and *Phlomoides* Moench with nine taxa. The remaining genera are represented by one to seven taxa (Fig. 2B).

**Figure 3.**
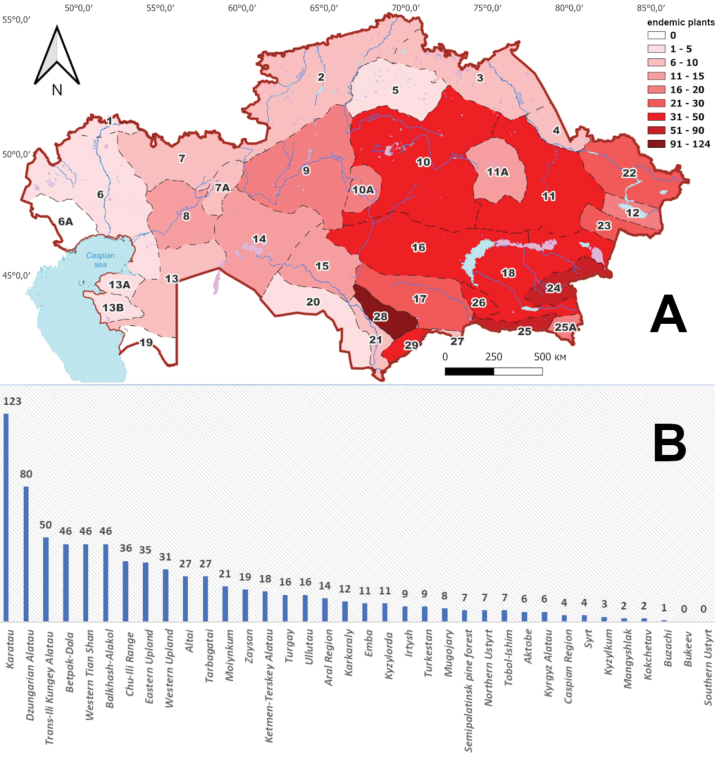
Endemic taxa richness in the floristic regions of Kazakhstan (**A**). The number of endemic taxa in the floristic regions of Kazakhstan (**B**).

The highest concentration of endemic plants was documented in two floristic regions: Karatau, with 123 taxa and Dzungarian Alatau, with 80 taxa. Additionally, a noteworthy range of 30 to 52 endemic taxa were observed in eight other floristic regions, namely Trans-Ili Kungey Alatau (50 taxa), Betpak-Dala (46 taxa), Western Tian Shan (46 taxa), Balkhash-Alakol (46 taxa), Chu-Ili Range (36 taxa), Eastern Upland (35 taxa), Western Upland (31 taxa) and Altai (27 taxa). On the other hand, a comparatively smaller number of endemic plants (not exceeding 5 taxa) were identified in six floristic regions: Caspian Region (4 taxa), Syrt (4 taxa), Kyzylkum (3 taxa), Mangyshlak (2 taxa), Kokchetav (2 taxa) and Buzachi (1 taxon). In the other two floristic regions (Bukeev, Southern Ustyrt), no endemic plants were found (Fig. 3A, B).

Amongst all endemic plants in Kazakhstan, 107 species, constituting 23.7% of the overall number of endemic taxa, are presently under the state-level legal protection (Baitulin 2014).

Amongst the six groups of life forms accepted by the Flora of Kazakhstan (Pavlov 1956), herbs include the greatest part of endemic taxa (367), followed by dwarf semishrubs (25 taxa), subshrubs (20 taxa), shrubs (23 taxa), undershrubs (13 taxa) and trees (3 taxa). The dominant life cycles are perennials (408 taxa), followed by annuals (33 taxa) and biennials (10 taxa).

According to the results of our research, 341 taxa previously considered endemics are recognised as sub-endemics of Kazakhstan (see Suppl. material 1) because they were found in the neighbouring countries. The largest number of plants previously considered endemic to Kazakhstan was found in China – 152 taxa, Kyrgyzstan – 138 taxa, Uzbekistan – 71 taxa, Russia – 59 taxa, Mongolia – 59 taxa, Tajikistan – 31 taxa, Turkmenistan – 19 taxa. The total of 169 former endemic taxa of Kazakhstan were synonymised to taxa with wider distribution ranges (see Suppl. material 2).

## ﻿Discussion

Based on our critical evaluation of vascular plants of Kazakhstan, 451 taxa are identified as endemic to the country (Appendix 1). This figure corresponds to 55–63% of the previously-reported numbers, i.e. 709–823 species (Pavlov 1956–1966; Bykov 1966; Goloskokov 1969; Baitenov 2001; Gemedjieva et al. 2010). The substantial difference in the number of endemic taxa compared to earlier publications is due to their reliance on outdated information solely derived from the nine-volume edition of the Flora of Kazakhstan (Pavlov 1956–1966). Our review reveals that 341 taxa previously considered endemics are to be treated as sub-endemics of Kazakhstan (see Suppl. material 1), whereas 169 former endemic taxa were synonymised to taxa with wider distribution ranges (see Suppl. material 2). For example, two most recent publications removed two endemic taxa from Kazakhstan: *Alliumvalentinae* Pavlov was found in Kyrgyzstan (Sennikov and Lazkov 2023), whereas the generic status of *Pseudomarrubium* was rejected (Zhao et al. 2023). Such examples provide evidence of ongoing taxonomic and floristic studies that are constantly shaping the list of endemic plants of Kazakhstan.

Despite the extensive territory and diverse natural conditions of Kazakhstan, the occurrence of endemic taxa appears highly uneven in the country. The largest number of endemic plants is concentrated in mountainous areas, specifically in the southern and south-eastern parts of Kazakhstan, within the following floristic regions: Karatau (123 taxa), followed by the Dzungarian Alatau (80 taxa) and Trans-Ili Kungey Alatau (50 taxa). These results fully align with the analysis presented by Gemedjieva et al. (2010), who examined the distributions of endemic plants in Kazakhstan, despite their use of outdated taxonomic and distributional data. The distribution pattern of endemic taxa across the territory of Kazakhstan supports the assertion made by Körner (2002) that mountain systems serve as biodiversity and endemism hotspots due to the compression of distinct climatic zones over varying altitudes. According to the latest global analysis of seed plant endemism, the territory of Kazakhstan is assigned to the centre of neo-endemism (Cai et al. 2023). The mountainous regions of Tian Shan in the south and the Altai Mountains in eastern Kazakhstan appear to have favoured this, because the mountainous regions exhibit a great diversity in their plant lineages and, therefore, contribute to high levels of neo-endemism.

The distribution of endemic plants in Kazakhstan is presumably influenced by the geological and climatic history of the territory. Past climate change and geological history help to explain how diversification and relictualisation shape the distribution of neo- and paleoendemism and simultaneously phylogenetic endemism worldwide (Cai et al. 2023). The influence of geological history on speciation, differentiation, migration and extinction of species has been highlighted by many scientists (Takhtajan 1969; Raven and Axelrod 1974; Latham and Ricklefs 1993; Axelrod et al. 1996; Huang et al. 2011). A prime example illustrating this phenomenon is the endemic taxa richness in the ancient Karatau Mountains (123 taxa), located at the westernmost limit of the Tian Shan. The Karatau Mountains possess a complex geological composition, characterised by the presence of the oldest Precambrian shale formations in Central Asia, as well as the Lower Paleozoic formations consisting of metamorphosed limestones and shales, overlain by a quartzite stratum (Kamelin 1990). An important factor in the distribution of endemic plants is long-term climatic stability. Central Asia is known for the presence of ancient plant lineages which survived there due to the continuous history of suitable climatic conditions (e.g. in *Lactuca* s.l.: Kilian et al. (2017)). The influence of geological and climatic history on the distribution of endemic plants in Kazakhstan requires additional research, given the poorly-studied bedrock types in Central Asia.

In the flora of Kazakhstan, herbs include most of the endemic taxa. The life forms of plants reflect their adaptability to environmental conditions and form the units of ecological classification, grouping plants with similar adaptive structures (Aipeisova 2009). According to Yurtsev (1976) and Rabotnov (1978), studies of life forms contribute to the understanding of species biology and their roles within ecosystems. The diversity of life forms represents a cumulative effect of long-term evolutionary processes responding to gradual changes in regional ecological conditions (Keller 1938; Shennikov 1950; Serebryakov 1964).

Amongst endemic plants of Kazakhstan, perennials (408 taxa) are most numerous. A global analysis of the distribution of plant life cycles around the world has shown that annual plants predominate in hot and arid conditions, especially during the long dry season (Poppenwimer et al. 2022). The number of annuals and biennials in Kazakhstan is 43 taxa or 9% of the total number of endemic plants of Kazakhstan. The distribution of endemic annuals over the territory of Kazakhstan is relatively uniform. The greatest number of annuals is noted in Western Upland (7 taxa), Zaysan (6 taxa) and Karatau (6 taxa). Amongst the families, the greatest number of annual endemic taxa is registered in Boraginaceae Juss. (17 taxa), Brassicaceae Burnett (8 taxa) and Amaranthaceae Juss. (6 taxa); in other families, 1–3 taxa are registered.

Given that numerous endemic plant species have restricted distributions, which makes them more prone to extinction (Myers et al. 2000; Pitman and Jørgensen 2002), it is crucial to emphasise the assessment and protection of such species (Baasanmunkh et al. 2022). Amongst 451 endemic taxa of Kazakhstan, 107 species are currently under state protection. We consider it necessary to further re-assess the status of protection of endemic plants of Kazakhstan using IUCN criteria.

During the critical examination of endemic plants in Kazakhstan, we found that some endemic plants were inaccurately attributed to other countries in the Plants of the World Online (POWO 2023). For instance, *Arthrophytumsubulifolium* Schrenk and *Atriplexiljinii* Aellen, according to POWO, are supposedly present in Turkmenistan. However, *Arthrophytumsubulifolium* Schrenk exclusively grows in a narrow region of the Chu-Ili Range in Kazakhstan (Osmanali et al. 2019), while *Atriplexiljinii* is solely found in the Mangistau, Aktobe and Kyzylorda Regions of Kazakhstan (Suchorukow 2007). *Stipaargillosa* Kotukhov and *Thalictrumbykovii* Kotukhov, along with *Gageaazutavica* Kotukhov, are incorrectly recorded in POWO as species native to the Altai Republic in the Russian Federation, whereas these species were described from East Kazakhstan (Kotukhov 1989, 1990, 1998).

At the same time, in the POWO database, certain species were erroneously assigned to Kazakhstan. For instance, the distributions of *Kameliniatianschanica* F.O.Khass. & I.I. Malzev, *Cousiniaxanthiocephala* Tscherneva, *Vicoakrascheninnikovii* Kamelin, *Phlomoidestschimganica* (Vved.) Adylov, Kamelin & Makhm. and *Erysimumaksaricum* Pavlov are limited to Uzbekistan (Kamelin 1976; Kupriyanov 2018; Tojibaev et al. 2020b). *Cousiniabalchanica* Tscherneva and *Minuartiapalyzanica* Proskur. were described from Turkmenistan (Proskuryakov 1987; Cherneva 1996), while *Taraxacumdarschajense* Orazova and *Hedysarumovczinnikovii* Karimova ex Kovalevsk. were reported to occur in Tajikistan (Orazova 1982; Adylov 1983) and *Crucianellaschischkinii* Lincz. was found in both Uzbekistan and Tajikistan (Kamelin 2017). These errors are associated with the difficulty of matching administrative boundaries with plant distribution areas in complicated mountainous territories.

## ﻿Conclusions

This checklist includes all strictly endemic plants of Kazakhstan, consisting of 451 taxa (species or subspecies) belonging to 139 genera and 34 families. The largest number of endemic taxa is concentrated in mountainous areas, specifically in the southern and south-eastern parts of Kazakhstan.

This paper serves as a fundamental groundwork for prospective investigations aimed at assessing population sizes and numbers of endemic taxa throughout Kazakhstan, crucial for determining their conservation status. Of course, this checklist of plant endemics of Kazakhstan is not final and will be revised in the future as a result of ongoing taxonomic and floristic studies.

## ﻿Additional information

### Author ORCIDs

Serik A. Kubentayev https://orcid.org/0000-0002-0369-0591

Daniyar T. Alibekov https://orcid.org/0000-0003-1555-1430

Yuri V. Perezhogin https://orcid.org/0000-0001-6997-8347

Georgy A. Lazkov https://orcid.org/0000-0002-3531-8524

Andrey N. Kupriyanov https://orcid.org/0000-0001-5602-2012

Alexander L. Ebel https://orcid.org/0000-0002-7889-4580

Klara S. Izbastina https://orcid.org/0000-0002-6418-1950

Olga V. Borodulina https://orcid.org/0000-0002-6080-716X

Balsulu B. Kubentayeva https://orcid.org/0000-0002-0841-6516

### Data availability

All of the data that support the findings of this study are available in the main text or Supplementary Information.

## ﻿Appendix 1. Annotated checklist of endemic taxa of Kazakhstan

In the checklist, families are listed in alphabetical order; lower-level taxa (genera, species and subspecies) within a family are also listed in alphabetical order.

The following information is given after the name of each taxon:

Life form (Lf) and life cycle (Lc) according to the Flora of Kazakhstan (Pavlov 1956); conservation status (Cs) according to the Red Book of Kazakhstan (Baitulin 2014). The species included in the Red Book of Kazakhstan are denoted by RB.

The distribution (D) of each taxon in Kazakhstan is given according to the floristic division of Kazakhstan (Pavlov 1956), where the territory of the country is divided into 29 floristic regions and seven subregions: 1 – Syrt, 2 – Tobol-Ishim, 3 – Irtysh, 4 – Semipalatinsk pine forest, 5 – Kokchetav, 6 – Caspian Region, 6a – Bukeev, 7 – Aktobe, 7a – Mugojary, 8 – Emba, 9 – Turgay, 10 – Western Upland, 10a – Ulutau, 11 – Eastern Upland, 11a – Karkaraly, 12 – Zaysan, 13 – Northern Ustyrt, 13a – Buzachi, 13b – Mangyshlak, 14 – Aral Region, 15 – Kyzylorda, 16 – Betpak-Dala, 17 – Moiynkum, 18 – Balkhash-Alakol, 19 – Southern Ustyrt, 20 – Kyzylkum, 21 – Turkestan, 22 – Altai, 23 – Tarbagatai, 24 – Dzungarian Alatau, 25 – Trans-Ili Kungey Alatau, 25a – Ketmen-Terskey Alatau, 26 – Chu-Ili Range, 27 – Kyrgyz Alatau, 28 – Karatau, 29 – Western Tian Shan.

Asterisks refer to annotations placed after the checklist.

**Fam. 1. Amaranthaceae Juss.**

**Gen. 1. *Anabasis* L.**

1. *Anabasisgypsicola* Iljin [*Lf*: Shrub. *Lc*: Per. *D*: 13, 16, 28]

2. *Anabasisturgaica* Iljin & Krasch. [*Lf*: Herb. *Lc*: Per. *Cs*: RB. *D*: 10a]

**Gen. 2. *Arthrophytum* Schrenk**

3. *Arthrophytumbalchaschense* (Iljin) Botsch. [*Lf*: Subshrub. *Lc*: Per. *D*: 16, 17, 18, 25]

4. *Arthrophytumbetpakdalense* Korovin & Mironov [*Lf*: Dwarf semishrub. *Lc*: Per. *D*: 16]

5. *Arthrophytumpulvinatum* Litv. [*Lf*: Dwarf semishrub. *Lc*: Per. *D*: 14]

6. *Arthrophytumsubulifolium* Schrenk * [*Lf*: Subshrub. *Lc*: Per. *D*: 26]

**Gen. 3. *Atriplex* L.**

7. *Atriplexiljinii* Aellen * [*Lf*: Herb. *Lc*: An. *D*: 8, 13, 14]

**Gen. 4. *Climacoptera* Botsch.**

8. *Climacopteraturgaica* (Iljin) Botsch. [*Lf*: Herb. *Lc*: An. *D*: 2, 6, 9, 14]

**Gen. 5. *Halimocnemis* C.A.Mey.**

9. *Halimocnemismironovii* Botsch. [*Lf*: Herb. *Lc*: An. *D*: 16, 26]

**Gen. 6. *Horaninovia* Fisch. & C.A.Mey.**

10. *Horaninoviacapitata* Sukhor. [*Lf*: Herb. *Lc*: An. *D*: 18]

**Gen. 7. *Nanophyton* Less.**

11. Nanophytonerinaceumsubsp.karataviense U.P.Pratov [*Lf*: Subshrub. *Lc*: Per. *D*: 28]

**Gen. 8. *Petrosimonia* Bunge**

12. *Petrosimoniahirsutissima* (Bunge) Iljin ex Pavlov [*Lf*: Herb. *Lc*: An. *D*: 9, 13, 15, 16, 17, 18]

**Gen. 9. *Salsola* L.**

13. *Salsolaeuryphylla* Botsch. [*Lf*: Undershrub. *Lc*: Per.*Cs*: RB. *D*: 14]

**Gen. 10. *Suaeda* Forssk. ex J.F.Gmel.**

14. *Suaedascabra* Lomon. [*Lf*: Herb. *Lc*: An. *D*: 14]

**Fam. 2. Amaryllidaceae J.St.-Hil.**

**Gen. 11. *Allium* L.**

15. *Alliumazutavicum* Kotukhov [*Lf*: Herb. *Lc*: Per. *D*: 22]

16. *Alliumbajtulinii* Bajtenov & I.I.Kamenetskaya [*Lf*: Herb. *Lc*: Per. *D*: 25]

17. *Alliumgoloskokovii* Vved. [*Lf*: Herb. *Lc*: Per. *D*: 23, 24]

18. *Alliumivasczenkoae* Kotukhov [*Lf*: Herb. *Lc*: Per. *D*: 22]

19. *Alliumiliense* Regel [*Lf*: Herb. *Lc*: Per. *D*: 26]

20. *Alliumjaxarticum* Vved. [*Lf*: Herb. *Lc*: Per. *D*: 29]

21. *Alliumkasteki* Popov. [*Lf*: Herb. *Lc*: Per. *Cs*: RB. *D*: 25]

22. *Alliumkoksuense* R.M. Fritsch, N. Friesen & S.V. Smirn. [*Lf*: Herb. *Lc*: Per. *D*: 24]

23. *Alliumkujukense* Vved. [*Lf*: Herb. *Lc*: Per. *D*: 28, 29]

24. *Alliumlasiophyllum* Vved. [*Lf*: Herb. *Lc*: Per. *D*: 25,25a]

25. *Alliumlehmannianum* Merckl. ex Bunge [*Lf*: Herb. *Lc*: Per. *D*: 9, 10, 15, 16, 20]

26. *Alliumlepsicum* R.M.Fritsch, N.Friesen & S.V.Smirn. [*Lf*: Herb. *Lc*: Per. *D*: 24]

27. *Alliumoreoprasoides* Vved. [*Lf*: Herb. *Lc*: Per. *D*: 28]

28. *Alliumsergii* Vved. [*Lf*: Herb. *Lc*: Per. *Cs*: RB. *D*: 28]

29. *Alliumscrobiculatum* Vved. [*Lf*: Herb. *Lc*: Per. *D*: 16, 17, 26]

30. *Alliumsubscabrum* (Regel) R.M.Fritsch [*Lf*: Herb. *Lc*: Per. *D*: 18, 26]

31. *Alliumtoksanbaicum* N.Friesen & Veselova [*Lf*: Herb. *Lc*: Per. *D*: 24]

32. *Alliumturtschicum* Regel [*Lf*: Herb. *Lc*: Per. *Cs*: RB. *D*: 28]

33. *Alliumvictoris* Vved. [*Lf*: Herb. *Lc*: Per. *D*: 29]

34. *Alliumviridulum* Ledeb. [*Lf*: Herb. *Lc*: Per. *D*: 11, 18, 23]

35. *Alliumzaissanicum* Kotukhov [*Lf*: Herb. *Lc*: Per. *D*: 12]

**Fam. 3. Apiaceae Lindl.**

**Gen. 12. *Autumnalia* Pimenov**

36. *Autumnaliabotschantzevii* Pimenov [*Lf*: Herb. *Lc*: Per. *D*: 28, 29]

**Gen. 13. *Eryngium* L.**

37. *Eryngiumkaratavicum* Iljin [*Lf*: Herb. *Lc*: Per. *Cs*: RB. *D*: 28]

**Gen. 14. *Ferula* L.**

38. *Ferulaglaberrima* Korovin [*Lf*: Herb. *Lc*: Per. *Cs*: RB. *D*: 17]

39. *Ferulagypsacea* Korovin [*Lf*: Herb. *Lc*: Per. *Cs*: RB. *D*: 21]

40. *Ferulaleucographa* Korovin * [*Lf*: Herb. *Lc*: Per. *Cs*: RB. *D*: 28]

41. *Ferulamalacophylla* Pimenov & J.V.Baranova [*Lf*: Herb. *Lc*: Per. *Cs*: RB. *D*: 28]

42. *Ferulapachyphylla* Korovin [*Lf*: Herb. *Lc*: Per. *D*: 28]

43. *Ferulasugatensis* Bajtenov [*Lf*: Herb. *Lc*: Per. *Cs*: RB. *D*: 25]

44. *Ferulataucumica* Baitenov [*Lf*: Herb. *Lc*: Per. *Cs*: RB. *D*: 18]

45. *Ferulaxeromorpha* Korovin [*Lf*: Herb. *Lc*: Per. *Cs*: RB. *D*: 21]

**Gen. 15. *Hyalolaena* Bunge**

46. *Hyalolaenatschuiliensis* (Pavlov) Pimenov & Kljuykov [*Lf*: Herb. *Lc*: Per. *Cs*: RB. *D*: 26]

**Gen. 16. *Karatavia* Pimenov & Lavrova**

47. *Karataviakultiassovii* (Korovin) Pimenov & Lavrova [*Lf*: Herb. *Lc*: Per. *Cs*: RB. *D*: 28, 29]

**Gen. 17. *Pachypleurum* Ledeb.**

48. *Pachypleurumaltaicum* Revuschkin [*Lf*: Herb. *Lc*: Per. *D*: 22]

**Gen. 18. *Pilopleura* Schischk.**

49. *Pilopleuragoloskokovii* (Korovin) Pimenov [*Lf*: Herb. *Lc*: Per. *Cs*: RB. *D*: 24]

**Gen. 19. *Prangos* Lindl.**

50. *Prangosdzhungarica* Pimenov [*Lf*: Herb. *Lc*: Per. *D*: 24]

51. *Prangosequisetoides* Kuzjmina [*Lf*: Herb. *Lc*: Per. *Cs*: RB. *D*: 28]

52. *Prangoslachnantha* (Korovin) Pimenov & Kljuykov [*Lf*: Herb. *Lc*: Per. *Cs*: RB. *D*: 17]

53. *Prangosmulticostata* Kljuykov & Lyskov * [*Lf*: Herb. *Lc*: Per. *D*: 23]

**Gen. 20. *Schrenkia* Fisch. & C.A.Mey.**

54. *Schrenkiacongesta* Korovin [*Lf*: Herb. *Lc*: Per. *D*: 28, 29]

55. *Schrenkiainvolucrata* Regel & Schmalh. [*Lf*: Herb. *Lc*: Per. *D*: 16, 17, 26, 28, 29]

56. *Schrenkiakultiassovii* Korovin [*Lf*: Herb. *Lc*: Per. *Cs*: RB. *D*: 29]

57. *Schrenkiapapillaris* Regel & Schmalh. [*Lf*: Herb. *Lc*: Per. *D*: 28]

**Gen. 21. *Schtschurowskia* Regel & Schmalh.**

58. *Schtschurowskiamargaritae* Korovin [*Lf*: Herb. *Lc*: Per. *Cs*: RB. *D*: 28]

**Gen. 22. *Seseli* L.**

59. *Seselibetpakdalense* Bajtenov [*Lf*: Herb. *Lc*: Per. *D*: 16]

60. *Seselimironovii* (Korovin) Pimenov & Sdobnina [*Lf*: Herb. *Lc*: Per. *D*: 16]

**Gen. 23. *Sphaenolobium* Pimenov**

61. *Sphaenolobiumkorovinii* Pimenov & Kljuykov [*Lf*: Herb. *Lc*: Per. *D*: 28, 29]

**Gen. 24. *Tschulaktavia* Bajtenov ex Pimenov & Kljuykov**

62. *Tschulaktaviasaxatilis* (Bajtenov) Bajtenov ex Pimenov & Kljuykov [*Lf*: Herb. *Lc*: Per. *Cs*: RB. *D*: 24]

**Fam. 4. Asparagaceae Juss.**

**Gen. 25. *Asparagus* Tourn. ex L.**

63. *Asparagusvvedenskyi* Botsch. [*Lf*: Herb. *Lc*: Per. *Cs*: RB. *D*: 21]

**Fam. 5. Asteraceae Bercht. & J.Presl**

**Gen. 26. *Alfredia* Cass.**

64. *Alfrediaintegrifolia* (Iljin) Tulyag. [*Lf*: Herb. *Lc*: Per. *D*: 24]

**Gen. 27. *Amberboa* (Pers.) Less.**

65. *Amberboatakhtajanii* Gabrieljan [*Lf*: Herb. *Lc*: An. *D*: 12]

**Gen. 28. *Arctium* L.**

66. *Arctiumalberti* (Regel & Schmalh.) S.López, Romasch., Susanna & N.Garcia [*Lf*: Herb. *Lc*: Per. *D*: 28, 29]

67. *Arctiumarctiodes* (Schrenk) Kuntze [*Lf*: Herb. *Lc*: Per. *D*: 9, 10, 15, 16]

68. *Arctiumgrandifolium* (Kult.) S.López, Romasch., Susanna & N.Garcia RB. [*Lf*: Herb. *Lc*: Per. *Cs*: RB. *D*: 28, 29]

69. *Arctiumugamense* (Karmysch.) S.López, Romasch., Susanna & N.Garcia [*Lf*: Herb. *Lc*: Per. *D*: 29]

**Gen. 29. *Artemisia* L.**

70. *Artemisiaaralensis* Krasch. [*Lf*: Dwarf semishrub. *Lc*: Per. *D*: 7, 9, 10, 14]

71. *Artemisiacamelorum* Krasch. [*Lf*: Dwarf semishrub. *Lc*: Per. *D*: 7, 7а, 8, 9, 10, 14, 15]

72. *Artemisiafilatovae* Kupr. [*Lf*: Dwarf semishrub. *Lc*: Per. *D*: 10]

73. *Artemisiahippolyti* A.Butkov [*Lf*: Dwarf semishrub. *Lc*: Per. *D*: 16]

74. *Artemisiakasakorum* (Krasch.) Pavlov [*Lf*: Herb. *Lc*: Per. *D*: 8, 11]

75. *Artemisiakotuchovii* Kupr. [*Lf*: Herb. *Lc*: Per. *D*: 22]

76. *Artemisiaquinqueloba* Trautv. [*Lf*: Subshrub. *Lc*: Per. *D*: 7, 8, 14, 15, 16]

77. *Artemisiasaurensis* Kupr. [*Lf*: Dwarf semishrub. *Lc*: Per. *D*: 23]

78. *Artemisiavalida* Krasch. ex Poljakov [*Lf*: Dwarf semishrub. *Lc*: Per. *D*: 21, 28]

**Gen. 30. *Brachanthemum* DC.**

79. *Brachanthemumkasakhorum* Krasch. [*Lf*: Dwarf semishrub. *Lc*: Per. *D*: 2, 10, 16, 18]

**Gen. 31. *Cancriniella* Tzvelev**

80. *Cancriniellakrascheninnikovii* (Rubtzov) Tzvelev [*Lf*: Herb. *Lc*: Per. *Cs*: RB. *D*: 16, 26]

**Gen. 32. *Centaurea* L.**

81. *Centaureakryloviana* Serg. * [*Lf*: Herb. *Lc*: Per. *D*: 11, 12, 22]

**Gen. 33. *Chondrilla* L.**

82. *Chondrillabosseana* Iljin [*Lf*: Herb. *Lc*: Per. *D*: 17, 18]

83. *Chondrillamacra* Iljin [*Lf*: Herb. *Lc*: Per. *D*: 16, 17]

84. *Chondrillamujunkumensis* Iljin & Igolkin [*Lf*: Herb. *Lc*: Per. *D*: 17, 18]

**Gen. 34. *Cousinia* Cass.**

85. *Cousiniaaspera* (Kult.) Karmysch. [*Lf*: Herb. *Lc*: Per. *D*: 29]

86. *Cousiniagomolitzkii* Juz. ex Tscherneva [*Lf*: Herb. *Lc*: Per. *D*: 28]

87. *Cousiniakasachstanica* Sennikov [*Lf*: Herb. *Lc*: Per. *D*: 27]

88. *Cousiniamindshelkensis* B.Fedtsch. [*Lf*: Herb. *Lc*: Per. *Cs*: RB. *D*: 28]

89. *Cousiniaperovskiensis* (Bornm.) Juz. ex Tschern. [*Lf*: Herb. *Lc*: Per. *D*: 14, 15, 16, 17, 18, 20, 24]

90. *Cousiniaschepsaica* Karmysch. [*Lf*: Herb. *Lc*: Per. *D*: 29]

91. *Cousiniaturkestanica* (Regel) Juz. [*Lf*: Herb. *Lc*: Per. *D*: 29]

92. *Cousiniaxanthiocephala* Tscherneva [*Lf*: Herb. *Lc*: Per. *D*: 21]

**Gen. 35. *Echinops* L.**

93. *Echinopskasakorum* Pavlov [*Lf*: Herb. *Lc*: Per. *Cs*: RB. *D*: 28]

94. *Echinopspubisquameus* Iljin [[*Lf*: Herb. *Lc*: Per. *D*: 28]

95. *Echinopssubglaber* Schrenk [*Lf*: Herb. *Lc*: Per. *D*: 15, 16, 28]

96. *Echinopstransiliensis* Golosk. [*Lf*: Herb. *Lc*: Per. *D*: 25, 26]

**Gen. 36. *Galatella* Cass.**

97. *Galatellabectauatensis* Kupr. & Koroljuk [*Lf*: Herb. *Lc*: Per. *D*: 11]

98. *Galatellapolygaloides* Novopokr. [*Lf*: Herb. *Lc*: Per. *D*: 25а]

99. *Galatellasaxatilis* Novopokr. [*Lf*: Herb. *Lc*: Per. *Cs*: RB. *D*: 25]

**Gen. 37. *Hieracium* L.**

100. *Hieraciumbectauatense* Kupr. [*Lf*: Herb. *Lc*: Per. *D*: 11]

**Gen. 38. *Jurinea* Cass.**

101. *Jurineaalmaatensis* Iljin [*Lf*: Herb. *Lc*: Per. *Cs*: RB. *D*: 25]

102. *Jurineabracteata* Regel & Schmalh. [*Lf*: Herb. *Lc*: Per. *D*: 24]

103. *Jurineacephalopoda* Iljin [*Lf*: Herb. *Lc*: Per. *Cs*: RB. *D*: 15, 21, 28]

104. *Jurineaczilikinoana* Iljin [*Lf*: Herb. *Lc*: Per. *D*: 28]

105. *Jurineaeximia* Tekutj. [*Lf*: Herb. *Lc*: Per. *Cs*: RB. *D*: 28]

106. *Jurineafedtschenkoana* Iljin [*Lf*: Herb. *Lc*: Per. *Cs*: RB. *D*: 1, 7, 7а, 10]

107. *Jurineahamulosa* Rubtzov [*Lf*: Herb. *Lc*: Per. *D*: 23, 24]

108. *Jurineakaratavica* Iljin [*Lf*: Herb. *Lc*: Per. *D*: 17, 18]

109. *Jurineakazachstanica* Iljin [*Lf*: Herb. *Lc*: Per. *D*: 6, 7, 8, 14]

110. *Jurineaknorringiana* Iljin [*Lf*: Herb. *Lc*: Per. *D*: 28]

111. *Jurineakrascheninnikovii* Iljin [*Lf*: Herb. *Lc*: Per. *D*: 9, 11а, 15, 16, 17, 26]

112. *Jurinealithophila* Rubtzov [*Lf*: Herb. *Lc*: Per. *D*: 24]

113. *Jurineamonticola* Iljin [*Lf*: Herb. *Lc*: Per. *D*: 28]

114. *Jurineamulticeps* Iljin [*Lf*: Herb. *Lc*: Per. *D*: 28]

115. *Jurineapineticola* Iljin [*Lf*: Herb. *Lc*: Per. *D*: 3, 4]

116. *Jurinearhizomatoidea* Iljin [*Lf*: Herb. *Lc*: Per. *D*: 28]

117. *Jurinearobusta* Schrenk [*Lf*: Herb. *Lc*: Per. *Cs*: RB. *D*: 16, 25, 26]

118. *Jurineaserratuloides* Iljin [*Lf*: Herb. *Lc*: Per. *D*: 12, 23]

119. *Jurineasuidunensis* Korsh. [*Lf*: Herb. *Lc*: Per. *D*: 12, 18, 24]

120. *Jurineaxerophytica* Iljin [*Lf*: Herb. *Lc*: Per. *D*: 4, 9, 11, 12, 14]

**Gen. 39. *Lamyropsis* (Kharadze) Dittrich**

121. *Lamyropsismacracantha* (Schrenk) Dittrich [*Lf*: Herb. *Lc*: Per. *D*:23, 24]

**Gen. 40. *Lepidolopha* C.Winkl.**

122. *Lepidolophagomolitzkii* Kovalevsk. & Safral. [*Lf*: Herb. *Lc*: Per. *D*: 28]

123. *Lepidolophakaratavica* Pavlov [*Lf*: Herb. *Lc*: Per. *Cs*: RB. *D*: 28]

124. *Lepidolophakrascheninnikovii* Czil. ex Kovalevsk. & Safral. [*Lf*: Herb. *Lc*: Per. *D*: 28]

125. *Lepidolophatalassica* Kovalevsk. & Safral. [*Lf*: Herb. *Lc*: Per. RB. *D*: 29]

**Gen. 41. *Rhaponticum* Vaill.**

126. *Rhaponticumkaratavicum* Regel & Schmalh. [*Lf*: Herb. *Lc*: Per. *Cs*: RB. *D*: 28]

**Gen. 42. *Ligularia* Cass.**

127. *Ligulariapavlovii* (Lipsch.) Cretz. [*Lf*: Herb. *Lc*: Per. *Cs*: RB. *D*: 28]

**Gen. 43. *Pseudoglossanthis* Poljakov**

128. *Pseudoglossanthisarctodshungarica* (Golosk.) Kamelin [*Lf*: Subshrub. *Lc*: Per. *Cs*: RB. *D*: 24]

129. *Pseudoglossanthissimulans* (Pavlov) Kamelin [*Lf*: Herb. *Lc*: Per. *D*: 29]

**Gen. 44. *Pseudopodospermum* (Lipsch. &Krasch.) Kuth.**

130. *Pseudopodospermumchantavicum* (Pavlov) Zaika, Sukhor. & N.Kilian [*Lf*: Herb. *Lc*: Per. *Cs*: RB. *D*: 26]

**Gen. 45. *Rhaponticoides* Vaill.**

131. *Rhaponticoideskultiassovii* (Iljin) Negaresh [*Lf*: Herb. *Lc*: Per. *Cs*: RB. *D*: 28]

132. *Rhaponticoidesphyllopoda* (Iljin) Negaresh [*Lf*: Herb. *Lc*: Per. *D*: 28, 29]

133. *Rhaponticoideszaissanica* Kupr., A.L. Ebel et Khrustaleva [*Lf*: Herb. *Lc*: Per. *D*: 12]

**Gen. 46. *Saussurea* DC.**

134. *Saussureamikeschinii* Iljin [*Lf*: Subshrub. *Lc*: Per. *Cs*: RB. *D*: 28]

135. *Saussureaninae* Iljin [*Lf*: Herb. *Lc*: Per. *D*: 24]

136. *Saussureapseudoblanda* Lipsch. ex Filat. [*Lf*: Herb. *Lc*: Per. *D*: 24]

**Gen. 47. *Scorzonera* L.**

137. *Scorzoneradianthoides* (Lipsch. & Krasch.) Lipsch. [*Lf*: Herb. *Lc*: Per. *D*: 11]

138. *Scorzonerafranchetii* Lipsch. [*Lf*: Herb. *Lc*: Per. *D*: 29]

139. *Scorzoneravavilovii* Kult. [*Lf*: Dwarf semishrub. *Lc*: Per. *D*: 28, 29]

**Gen. 48. *Senecio* L.**

140. *Senecioiljinii* Schischk. [*Lf*: Herb. *Lc*: Per. *D*: 24]

141. *Senecionuraniae* Roldugin [*Lf*: Herb. *Lc*: An. *D*: 29]

**Gen. 49. *Takhtajaniantha* Nazarova**

142. *Takhtajanianthaveresczaginii* (Kamelin & S.V.Smirn.) Zaika, Sukhor. & N.Kilian [*Lf.* Herb. *Lc*: Per. D: 22]

**Gen. 50. *Tanacetopsis* (Tzvelev) Kovalevsk.**

143. *Tanacetopsisgoloskokovii* (Poljakov) Karmysch. [*Lf*: Herb. *Lc*: Per. *Cs*: RB. *D*: 24, 25]

144. *Tanacetopsispjataevae* (Kovalevsk.) Karmysch. [*Lf*: Herb. *Lc*: Per. *Cs*: RB. *D*: 28]

145. *Tanacetopsispopovii* Kamelin & Kovalevsk. [*Lf*: Herb. *Lc*: Per. *D*: 28]

**Gen. 51. *Tanacetum* L.**

146. *Tanacetumcorymbiforme* (Tzvelev) K.Bremer & Humphries [*Lf*: Herb. *Lc*: Per. *D*: 23, 24]

147. *Tanacetumkelleri* (Krylov & Plotn.) Takht. [*Lf*: Herb. *Lc*: Per. *Cs*: RB. *D*: 22]

148. *Tanacetummindshelkense* Kovalevsk. [*Lf*: Herb. *Lc*: Per. *D*: 28]

149. *Tanacetumsaryarkense* Kamelin [*Lf*: Herb. *Lc*: Per. *D*: 16, 26]

150. *Tanacetumsaxicola* (Krasch.) Tzvelev [*Lf*: Herb. *Lc*: Per. *Cs*: RB. *D*: 7а, 8]

151. *Tanacetumulutavicum* Tzvelev [*Lf*: Herb. *Lc*: Per. *Cs*: RB. *D*: 10a]

**Gen. 52. *Taraxacum* F.H.Wigg.**

152. *Taraxacumalmaatense* Schischk. [*Lf*: Herb. *Lc*: Per. *D*: 25]

153. *Taraxacumarasanum* R.Doll [*Lf*: Herb. *Lc*: Per. *D*: 25]

154. *Taraxacumatrochlorinum* Kirschner & Štěpánek [*Lf*: Herb. *Lc*: Per. *D*: 25]

155. *Taraxacumbotschantzevii* Schischk. [*Lf*: Herb. *Lc*: Per. *D*: 28]

156. *Taraxacumcornucopiae* Kirschner & Štěpánek [*Lf*: Herb. *Lc*: Per. *D*: 25]

157. *Taraxacumcorvinum* Kirschner & Štěpánek [*Lf*: Herb. *Lc*: Per. *D*: 25]

158. *Taraxacumdzhungaricola* Kirschner & Štěpánek [*Lf*: Herb. *Lc*: Per. *D*: 24]

159. *Taraxacumglabellum* Schischk. [*Lf*: Herb. *Lc*: Per. *D*: 28]

160. *Taraxacumkaratavicum* Pavlov [*Lf*: Herb. *Lc*: Per. *D*: 28]

161. *Taraxacumkasachiforme* R.Doll [*Lf*: Herb. *Lc*: Per. *D*: 25]

162. *Taraxacumkasachum* R.Doll [*Lf*: Herb. *Lc*: Per. *D*: 25]

163. *Taraxacummagnum* Korol. [*Lf*: Herb. *Lc*: Per. *D*: 25a]

164. *Taraxacummedeense* R.Doll [*Lf*: Herb. *Lc*: Per. *D*: 24, 25]

165. *Taraxacumperpusillum* Schischk. [*Lf*: Herb. *Lc*: Per. *D*: 24]

166. *Taraxacumpseudolugubre* R.Doll [*Lf*: Herb. *Lc*: Per. *D*: 25]

167. *Taraxacumpseudotianschanicum* R.Doll [*Lf*: Herb. *Lc*: Per. *D*: 8]

168. *Taraxacumsaposhnikovii* Schischk. [*Lf*: Herb. *Lc*: Per. *D*: 23, 24]

169. *Taraxacumsublilacinum* Kirschner & Štěpánek [*Lf*: Herb. *Lc*: Per. *D*: 25]

170. *Taraxacumurdzharense* Orazova [*Lf*: Herb. *Lc*: Per. *D*: 23]

171. *Taraxacumviolaceum* R.Doll [*Lf*: Herb. *Lc*: Per. *D*: 25]

**Gen. 53. *Tragopogon* L.**

172. *Tragopogonkarelinii* S.A.Nikitin [*Lf*: Herb. *Lc*: Bi. *D*: 10, 16, 18, 23, 24]

**Gen. 54. *Vickifunkia* C.Ren, L.Wang, I.D.Illar. & Q.E.Yang**

173. *Vickifunkiakareliniana* (Stschegl.) C.Ren, L.Wang, I.D.Illar. & Q.E.Yang [*Lf*: Herb. *Lc*: Per. *D*: 23]

**Fam. 6. Berberidaceae Juss.**

**Gen. 55. *Berberis* L.**

174. *Berberiskarkaralensis* Kornil. & Potapov [*Lf*: Shrub. *Lc*: Per. *Cs*: RB. *D*: 11a]

**Fam. 7. Betulaceae Gray**

**Gen. 56. *Betula* L.**

175. *Betulakaragandensis* V.N.Vassil. [*Lf*: Tree. *Lc*: Per. *D*: 11а]

176. *Betulasaviczii* V.N.Vassil. [*Lf*: Tree. *Lc*: Per. *D*: 10]

**Fam. 8. Bignoniaceae Juss.**

**Gen. 57. *Incarvillea* Juss.**

177. *Incarvilleasemiretschenskia* (B.Fedtsch.) Grierson [*Lf*: Herb. *Lc*: Per. *Cs*: RB. *D*: 26]

**Fam. 9. Boraginaceae Juss.**

**Gen. 58. *Eritrichium* Schrad. ex Gaudin**

178. *Eritrichiumrelictum* Kudab. [*Lf*: Herb. *Lc*: Per. *D*: 24]

**Gen. 59. *Heliotropium* Tourn. ex L.**

179. *Heliotropiumparvulum* Popov [*Lf*: Herb. *Lc*: An. *Cs*: RB. *D*: 16, 18, 25]

**Gen. 60. *Lappula* Moench**

180. *Lappulabaitenovii* Kudab. [*Lf*: Herb. *Lc*: Bi. *D*: 25]

181. *Lappulacoronifera* Popov [*Lf*: Herb. *Lc*: An. *D*: 11]

182. *Lappulacristata* (Bunge) B.Fedtsch. *[*Lf*: Herb. *Lc*: An. *D*: 10, 11, 12]

183. *Lappuladiploloma* (Fisch. & C.A.Mey.) Gürke [*Lf*: Herb. *Lc*: An. *D*: 9, 11]

184. *Lappulaglabrata* Popov [*Lf*: Herb. *Lc*: Bi. *Cs*: RB. *D*: 16]

185. *Lappulaketmenica* Kudab. [*Lf*: Herb. *Lc*: An. *D*: 25a]

186. *Lappulakuprijanovii* Ovczinnikova [*Lf*: Herb. *Lc*: Bi. RB. *D*: 28]

187. *Lappulalipschitzii* Popov [*Lf*: Herb. *Lc*: An. *D*: 21]

188. *Lappulapavlovii* Golosk. [*Lf*: Herb. *Lc*: An. *D*: 24]

189. *Lappulasaphronovae* Kamelin [*Lf*: Herb. *Lc*: Bi. *D*: 13b]

190. *Lappulazaissanica* (Aralbaev) Aralbaev [*Lf*: Herb. *Lc*: An. *D*: 12]

**Gen. 61. *Lepechiniella* Popov**

191. *Lepechiniellaaustrodshungarica* Golosk. [*Lf*: Herb. *Lc*: An.-Bi. *D*: 10, 24]

192. *Lepechiniellamichaelis* Golosk. [*Lf*: Herb. *Lc*: Per. *Cs*: RB. *D*: 24]

193. *Lepechiniellaomphaloides* (Schrenk) Popov [*Lf*: Herb. *Lc*: Bi. *D*: 10]

194. *Lepechiniellasaurica* (Bajtenov & Kudab.) Ovczinnikova [*Lf*: Herb. *Lc*: An. *D*: 23]

**Gen. 62. *Mattiastrum* (Boiss.) Brand**

195. *Mattiastrumkarataviense* (Pavlov ex Popov) Czerep. [*Lf*: Herb. *Lc*: Per. *Cs*: RB. *D*: 28]

**Gen. 63. *Myosotis* L.**

196. *Myosotiskazakhstanica* O.D.Nikif. [*Lf*: Herb. *Lc*: An. *D*: 10, 10a, 11, 11a]

**Gen. 64. *Paracaryum* Boiss.**

197. *Paracaryumintegerrimum* Myrz. [*Lf*: Herb. *Lc*: Per. *Cs*: RB. *D*: 28]

**Gen. 65. *Rindera* Pall.**

198. *Rinderaochroleuca* Kar. & Kir. * [*Lf*: Herb. *Lc*: Per. *Cs*: RB. *D*: 18]

**Gen. 66. *Rochelia* Rchb.**

199. *Rochelialeiosperma* (Popov) Golosk. [*Lf*: Herb. *Lc*: An. *D*: 24]

**Gen. 67. *Sauria* Bajtenov**

200. *Sauriaakkolia* Bajtenov [*Lf*: Herb. *Lc*: Per. *D*: 17]

**Fam. 10. Brassicaceae Burnett**

**Gen. 68. *Botschantzevia* Nabiev**

201. *Botschantzeviakaratavica* (Lipsch. & Pavlov) Nabiev [*Lf*: Dwarf semishrub. *Lc*: Per. *Cs*: RB. *D*: 28]

**Gen. 69. *Clausia* Korn-Trotzky.**

202. *Clausiakasakorum* Pavlov [*Lf*: Herb. *Lc*: Per. *D*: 10a]

203. *Clausiarobusta* Pachom. [*Lf*: Herb. *Lc*: Per. *D*: 5]

**Gen. 70. *Erysimum* Tourn. ex L.**

204. *Erysimumkazachstanicum* Botsch. [*Lf*: Herb. *Lc*: Bi. *D*: 10, 10а, 11, 23]

**Gen. 71. *Eutrema* R.Br.**

205. *Eutremahalophilum* (C.A.Mey.) Al-Shehbaz & Warwick* [*Lf*: Herb. *Lc*: An. *D*: 2, 3, 4, 11, 12]

206. *Eutremaplatypetalum* (Schrenk) Al-Shehbaz & Warwick [*Lf*: Herb. *Lc*: Per. *D*: 24]

**Gen. 72. *Isatis* Tourn. ex L.**

207. *Isatiscanaliculata* (Vassilcz.) V.V.Botschantz. [*Lf*: Herb. *Lc*: Bi. *D*: 9, 10]

208. *Isatisdeserti* (N.Busch) V.V.Botschantz. [*Lf*: Herb. *Lc*: An. *D*: 16]

**Gen. 73. *Lepidium* L.**

209. *Lepidiumjarmolenkoi* V.M.Vinogr. [*Lf*: Herb. *Lc*: Per. *D*: 16]

210. *Lepidiumkarataviense* Regel & Schmalh. [*Lf*: Herb. *Lc*: Per. *D*: 28]

211. *Lepidiummummenhoffianum* Al-Shehbaz [*Lf*: Herb. *Lc*: Per. *D*: 24]

212. *Lepidiumpavlovii* Al-Shehbaz & Mummenhoff [*Lf*: Herb. *Lc*: Per. *Cs*: RB. *D*: 28, 29]

213. *Lepidiumrobustum* (Pavlov) Al-Shehbaz [*Lf*: Herb. *Lc*: Per. *Cs*: RB. *D*: 28]

214. *Lepidiumsagittatum* (Kar. & Kir.) Al-Shehbaz [*Lf*: Herb. *Lc*: Per. *Cs*: RB. *D*: 23, 24]

215. *Lepidiumtrautvetteri* (Botsch.) Al-Shehbaz [*Lf*: Herb. *Lc*: Per. *Cs*: RB. *D*: 16, 18]

**Gen. 74. *Parrya* R.Br.**

216. *Parryalongicarpa* Krasn. [*Lf*: Herb. *Lc*: Per. *D*: 26]

217. *Parryapapillosa* (Vassilcz.) D.A.German & Al-Shehbaz [*Lf*: Herb. *Lc*: An. *D*: 28, 29]

218. *Parryapavlovii* A.N.Vassiljeva [*Lf*: Herb. *Lc*: Per. *D*: 28]

219. *Parryapazijae* (Pachom.) D.A.German & Al-Shehbaz [*Lf*: Undershrub. *Lc*: Per. *D*: 28, 29]

220. *Parryasaurica* (Pachom.) D.A.German & Al-Shehbaz [*Lf*: Herb. *Lc*: Per. *D*: 23]

221. *Parryavvedenskyi* (Pachom.) D.A.German & Al-Shehbaz [*Lf*: Herb. *Lc*: Bi. *D*: 28, 29]

**Gen. 75. *Rhammatophyllum* O.E.Schulz**

222. *Rhammatophyllumpachyrhizum* (Kar. & Kir.) O.E.Schulz [*Lf*: Dwarf semishrub. *Lc*: Per. *D*: 7а, 8, 9, 10, 10а, 11, 13, 14, 16, 22, 24]

**Gen. 76. *Strigosella* Boiss.**

223. *Strigosellamyrzakulovii* Bajtenov [*Lf*: Herb. *Lc*: An. *D*: 28, 29]

**Fam. 11. Campanulaceae Juss.**

**Gen. 77. *Sergia* Fed.**

224. *Sergiasewerzowii* (Regel) Fed. [*Lf*: Herb. *Lc*: Per. *D*: 28, 29]

**Fam. 12. Caryophyllaceae Juss.**

**Gen. 78. *Eremogone* Fenzl**

225. *Eremogoneturlanica* (Bajtenov) Czerep. [*Lf*: Herb. *Lc*: Per. *Cs*: RB. *D*: 28]

**Gen. 79. *Dianthus* L.**

226. *Dianthuskarataviensis* Pavlov [*Lf*: Herb. *Lc*: Per. RB. *D*: 28, 29]

227. *Dianthusmultisquameus* Bondarenko & R.M.Vinogr. [*Lf*: Herb. *Lc*: Per. *D*: 28, 29]

**Gen. 80. *Gypsophila* L.**

228. *Gypsophilaaulieatensis* B.Fedtsch. [*Lf*: Herb. *Lc*: Per. *Cs*: RB. *D*: 28]

**Gen. 81. *Silene* L.**

229. *Sileneanisoloba* Schrenk [*Lf*: Herb. *Lc*: Per. *D*: 10, 10a]

230. *Silenebetpakdalensis* Bajtenov [*Lf*: Herb. *Lc*: Per. *Cs*: RB. *D*: 13, 16, 26]

231. *Silenejaxartica* Pavlov [*Lf*: Herb. *Lc*: Per. *Cs*: RB. *D*: 28]

232. *Silenemuslimii* Pavlov [*Lf*: Herb. *Lc*: Per. *Cs*: RB. *D*: 24, 26]

**Fam. 13. Convolvulaceae Juss.**

**Gen. 82. *Cuscuta* L.**

233. *Cuscutacamelorum* Pavlov [*Lf*: Herb. *Lc*: An. *D*: 28]

234. *Cuscutaelpassiana* Pavlov [*Lf*: Herb. *Lc*: An. *D*: 26]

235. *Cuscutakaratavica* Pavlov [*Lf*: Herb. *Lc*: An. *D*: 28]

**Fam. 14. Crassulaceae J.St.-Hil.**

**Gen. 83. *Pseudosedum* (Boiss.) A.Berger**

236. *Pseudosedumkaratavicum* Boriss. [*Lf*: Herb. *Lc*: Per. *Cs*: RB. *D*: 28]

**Fam. 15. Cyperaceae Juss.**

**Gen. 84. *Cyperus* L.**

237. *Cyperussoongoricus* Kar. & Kir. [*Lf*: Herb. *Lc*: An. *D*: 12]

**Fam. 16. Euphorbiaceae Juss.**

**Gen. 85. *Euphorbia* L.**

238. *Euphorbiaheptapotamica* Golosk. [*Lf*: Herb. *Lc*: An. *D*: 24]

239. *Euphorbiakalbaensis* Baikov & I.V.Khan [*Lf*: Herb. *Lc*: Per. *D*: 22]

240. *Euphorbiasaurica* Baikov [*Lf*: Herb. *Lc*: Per. *D*: 23]

241. *Euphorbiayaroslavii* Poljakov [*Lf*: Herb. *Lc*: Per. *Cs*: RB. *D*: 25]

**Fam. 17. Fabaceae Lindl.**

**Gen. 86. *Astragalus* L.**

242. *Astragalusabbreviatus* Kar. & Kir. [*Lf*: Herb. *Lc*: Per. *D*: 24, 25, 25а, 26, 27]

243. *Astragalusarganaticus* Bunge [*Lf*: Herb. *Lc*: Per. *D*: 18, 24]

244. *Astragalusbalchaschensis* Sumnev. [*Lf*: Herb. *Lc*: Per. *D*: 18]

245. *Astragalusbrotherusii* Podlech [*Lf*: Herb. *Lc*: Per. *D*: 25]

246. *Astragaluschaetolobus* Bunge [*Lf*: Subshrub. *Lc*: Per. *D*: 4, 11, 22]

247. *Astragaluscitoinflatus* Bondarenko [*Lf*: Herb. *Lc*: Per. *D*: 17]

248. *Astragalusclausii* C.A.Mey. [*Lf*: Herb. *Lc*: Per. *D*: 6]

249. *Astragaluscytisoides* Bunge [*Lf*: Dwarf semishrub. *Lc*: Per. *D*: 18]

250. *Astragalusfragiformis* Willd. [*Lf*: Undershrub. *Lc*: Per. *D*: 22]

251. *Astragalusgeorgii* Gontsch. [*Lf*: Dwarf semishrub. *Lc*: Per. *D*: 28]

252. *Astragalusinflatus* DC. [*Lf*: Undershrub. *Lc*: Per. *D*: 22]

253. *Astragalusjaxarticus* Pavlov [*Lf*: Herb. *Lc*: Per. *D*: 26, 28]

254. *Astragalusjuvenalis* Delile [*Lf*: Herb. *Lc*: An. *D*: 10, 18]

255. *Astragaluskarataviensis* Pavlov [*Lf*: Dwarf semishrub. *Lc*: Per. *Cs*: RB. *D*: 21, 28]

256. *Astragaluskaratjubeki* Golosk. [*Lf*: Subshrub. *Lc*: Per. *D*: 16, 18]

257. Astragaluskasachstanicussubsp.coloratus Knjaz. [*Lf*: Herb. *Lc*: Per. *D*: 10, 11а]

258. *Astragaluskazymbeticus* Saposhn. ex Sumnev. [*Lf*: Herb. *Lc*: Per. *D*: 24]

259. *Astragaluskopalensis* Lipsky [*Lf*: Shrub. *Lc*: Per. *Cs*: RB. *D*: 24]

260. *Astragaluskrascheninnikovii* Kamelin [*Lf*: Shrub. *Lc*: Per. *Cs*: RB. *D*: 16]

261. *Astragaluskrasnovii* Popov [*Lf*: Herb. *Lc*: Per. *D*: 26]

262. *Astragalusleucocalyx* Popov * [*Lf*: Shrub. *Lc.* Per. *D*: 28]

263. *Astragaluslipschitzii* Pavlov [*Lf*: Undershrub. *Lc*: Per. *D*: 28, 29]

264. *Astragalusmokeevae* Popov [*Lf*: Subshrub. *Lc*: Per. *D*: 28]

265. *Astragalusneopopovii* Golosk. [*Lf*: Herb. *Lc*: Per. *D*: 24]

266. *Astragaluspsammophilus* Golosk. [*Lf*: Subshrub. *Lc*: Per. *D*: 18]

267. *Astragaluspseudocytisoides* Popov *Lf*: Dwarf semishrub. *Lc*: Per. *Cs*: RB. *D*: 25, 26]

268. *Astragaluspsilopus* Schrenk [*Lf*: Herb. *Lc*: Per. *D*: 18, 24]

269. *Astragaluspulposus* Popov [*Lf*: Herb. *Lc*: Per. *D*: 25]

270. *Astragaluspycnolobus* Bunge [*Lf*: Subshrub. *Lc*: Per. *D*: 12, 22]

271. *Astragalusrariflorus* Ledeb. * [*Lf*: Herb. *Lc*: Per. *D*: 11]

272. *Astragalusrubtzovii* Boriss. [*Lf*: Herb. *Lc*: Per. *Cs*: RB. *D*: 25а]

273. *Astragalussaphronovae* Kulikov [*Lf*: Dwarf semishrub. *Lc*: Per. *D*: 7, 13, 13b]

274. *Astragalussarchanensis* Gontsch. [*Lf*: Herb. *Lc*: Per. *D*: 24]

275. *Astragalussemenovii* Bunge [*Lf*: Herb. *Lc*: Per. *D*: 18, 24, 25а]

276. *Astragalussisyrodytes* Bunge [*Lf*: Herb. *Lc*: Per. *D*: 28]

277. *Astragalusspartioides* Kar. & Kir. [*Lf*: Subshrub. *Lc*: Per. *D*: 18]

278. *Astragalusspeciosissimus* Pavlov [*Lf*: Subshrub. *Lc*: Per. *D*: 28]

279. *Astragalussubcaracugensis* Sitpaeva [*Lf*: Subshrub. *Lc*: Per. *D*: 9]

280. *Astragalussubternatus* Pavlov [*Lf*: Herb. *Lc*: Per. *Cs*: RB. *D*: 28]

281. *Astragalussumneviczii* Pavlov [*Lf*: Herb. *Lc*: Per. *Cs*: RB. *D*: 16]

282. *Astragalusterektensis* Fisjun [*Lf*: Herb. *Lc*: Per. *D*: 24]

283. *Astragalustransnominatus* M.N.Abdull. [*Lf*: Herb. *Lc*: Per. *D*: 26, 28]

284. *Astragalustscharynensis* Popov [*Lf*: Undershrub. *Lc*: Per. *Cs*: RB. *D*: 24, 25]

285. *Astragalusturajgyricus* Golosk. [*Lf*: Herb. *Lc*: Per. *D*: 25]

286. *Astragalusunilateralis* Kar. & Kir. [*Lf*: Herb. *Lc*: Per. *D*: 4, 7, 8, 11, 12, 22]

287. *Astragalusvirens* Pavlov [*Lf*: Herb. *Lc*: Per. *D*: 28]

**Gen. 87. *Caragana* Lam.**

288. *Caraganamedia* Sanchir [*Lf*: Shrub. *Lc*: Per. *D*: 10, 11]

**Gen. 88. *Chesneya* Lindl. ex Endl.**

289. *Chesneyakaratavica* Kamelin [*Lf*: Herb. *Lc*: Per. *D*: 28]

**Gen. 89. *Hedysarum* L.**

290. *Hedysarumbectauatavicum* Bajtenov [*Lf*: Herb. Lc: Per. Cs: RB. D: 11]

291. *Hedysarumchantavicum* Popov ex Bajtenov [*Lf*: Herb. *Lc*: Per. *D*: 26]

292. *Hedysarumkarataviense* B.Fedtsch. [*Lf*: Herb. *Lc*: Per. *Cs*: RB. *D*: 28]

293. *Hedysarumnikolai* Kovalevsk. [*Lf*: Herb. *Lc*: Per. *D*: 28]

294. *Hedysarummindshilkense* Bajtenov [*Lf*: Herb. *Lc*: Per. *Cs*: RB. *D*: 28]

295. *Hedysarumpallidiflorum* Pavlov [*Lf*: Herb. *Lc*: Per. *D*: 28]

296. *Hedysarumpavlovii* Bajtenov [*Lf*: Herb. *Lc*: Per. *D*: 28]

297. *Hedysarumtarbagataicum* Knjaz. [*Lf*: Herb. *Lc*: Per. *D*: 3, 11, 12, 22, 23]

298. *Hedysarumulutavicum* Knjaz. [*Lf*: Herb. *Lc*: Per. *D*: 10а]

299. *Hedysarumvillosissimum* Knjaz. [*Lf*: Herb. *Lc*: Per. *D*: 10, 11]

**Gen. 90. *Onobrychis* Mill.**

300. *Onobrychisalatavica* Bajtenov * [*Lf*: Herb. *Lc*: Per. *Cs*: RB. *D*: 25]

**Gen. 91. *Oxytropis* DC.**

301. *Oxytropisalberti-regelii* Vassilcz. [*Lf*: Herb. *Lc*: Per. *D*: 29]

302. *Oxytropisalmaatensis* Bajtenov [*Lf*: Herb. *Lc*: Per. *Cs*: RB. *D*: 25, 25а]

303. *Oxytropisbajtulinii* Kotukhov [*Lf*: Herb. *Lc*: Per. *D*: 22]

304. *Oxytropisbiloba* Saposhn. [*Lf*: Herb. *Lc*: Per. *Cs*: RB. *D*: 23]

305. *Oxytropisbosculensis* Golosk. [*Lf*: Herb. *Lc*: Per. *D*: 25]

306. *Oxytropisbrevicaulis* Ledeb. [*Lf*: Herb. *Lc*: Per. *D*: 2, 3, 9, 10, 11, 18]

307. *Oxytropiscanopatula* Vassilcz. [*Lf*: Herb. *Lc*: Per. *D*: 28]

308. *Oxytropiscretacea* Basil. [*Lf*: Herb. *Lc*: Per. *D*: 1]

309. *Oxytropisechidna* Vved. [*Lf*: Undershrub. *Lc*: Per. *Cs*: RB. *D*: 28]

310. *Oxytropisfruticulosa* Bunge [*Lf*: Undershrub. *Lc*: Per. *D*: 24]

311. *Oxytropisgebleriana* Schrenk [*Lf*: Herb. *Lc*: Per. *D*: 1, 2, 3, 7а, 9, 10, 11, 16, 18]

312. *Oxytropisheteropoda* Bunge [*Lf*: Herb. *Lc*: Per. *D*: 24, 25]

313. *Oxytropiskarataviensis* Pavlov [*Lf*: Herb. *Lc*: Per. *Cs*: RB. *D*: 28]

314. *Oxytropiskyziltalensis* Vassilcz. [*Lf*: Herb. *Lc*: Per. *D*: 24]

315. *Oxytropisniedzweckiana* Popov [*Lf*: Herb. *Lc*: Per. *Cs*: RB. *D*: 25]

316. *Oxytropispulvinoides* Vassilcz. [*Lf*: Herb. *Lc*: Per. *D*: 24]

317. *Oxytropissatpaevii* Bajtenov [*Lf*: Herb. *Lc*: Per. *D*: 11]

318. *Oxytropissubcapitata* Gontsch [*Lf*: Herb. *Lc*: Per. *D*: 28]

319. *Oxytropissubverticillaris* C.A.Mey. [*Lf*: Herb. *Lc*: Per. *Cs*: RB. *D*: 3, 10, 10а, 11]

320. *Oxytropissumneviczii* Krylov [*Lf*: Herb. *Lc*: Per. *D*: 22]

321. *Oxytropistalgarica* Popov [*Lf*: Herb. *Lc*: Per. *D*: 25]

322. *Oxytropistomentosa* Gontsch. [*Lf*: Herb. *Lc*: Per. *D*: 28]

**Fam. 18. Frankeniaceae Desv.**

**Gen. 92. *Frankenia* L.**

323. Frankeniabucharicasubsp.mironovii (Botsch.) Chrtek [*Lf*: Subshrub. *Lc*: Per. *D*: 16, 25, 26]

324. Frankeniabucharicasubsp.transkaratavica (Botsch.) Chrtek [*Lf*: Subshrub. *Lc*: Per. *D*: 17]

**Fam. 19. Gentianaceae Juss.**

**Gen. 93. *Comastoma* Toyok.**

325. *Comastomairinae* (Pachom.) Czerep. [*Lf*: Herb. *Lc*: An. *D*: 25]

**Fam. 20. Lamiaceae Martinov**

**Gen. 94. *Dracocephalum* L.**

326. *Dracocephalumpavlovii* Roldugin [*Lf*: Dwarf semishrub. *Lc*: Per. *D*: 29]

**Gen. 95. *Phlomoides* Moench.**

327. *Phlomoidesaffinis* (Schrenk) Salmaki [*Lf*: Herb. *Lc*: Per. *D*: 10, 10а, 11, 16, 17, 18, 28]

328. *Phlomoidesboraldaica* A.L.Ebel [*Lf*: Herb. *Lc*: Per. *D*: 28, 29]

329. *Phlomoidesczuiliensis* (Golosk.) Adylov, Kamelin & Makhm. [*Lf*: Herb. *Lc*: Per. *D*: 26]

330. *Phlomoideseremostachydioides* (Popov) Y.Zhao & C.L.Xiang [*Lf*: Herb. *Lc: Cs*: RB. Per. *D*: 28]

331. *Phlomoidesgymnocalyx* (Schrenk) Adylov, Kamelin & Makhm. [*Lf*: Herb. *Lc*: Per. *D*: 18, 24, 26]

332. *Phlomoidesiliensis* (Regel) Adylov, Kamelin & Makhm [*Lf*: Herb. *Lc*: Per. *D*: 18, 24]

333. *Phlomoidespectinata* (Popov) Adylov, Kamelin & Makhm. [*Lf*: Herb. *Lc*: Per. *D*: 28]

334. *Phlomoidesrotala* (Schrenk ex Fisch., C.A.Mey. & Avé-Lall.) Salmaki [*Lf*: Herb. *Lc*: Per. *D*: 18]

335. *Phlomoidesseptentrionalis* (Popov) Adylov, Kamelin & Makhm. [*Lf*: Herb. *Lc*: Per. *D*: 27, 28, 29]

**Gen. 96. *Phlomis* L.**

336. *Phlomismindshelkensis* Lazkov [*Lf*: Herb. *Lc*: Per. *D*: 28]

**Gen. 97. *Lagochilus* Bunge ex Benth.**

337. *Lagochilusandrossowii* Knorring [*Lf*: Dwarf semishrub. *Lc*: Per. *D*: 15, 28]

338. *Lagochiluslongidentatus* Knorring [*Lf*: Dwarf semishrub. *Lc*: Per. *D*: 16, 28]

339. *Lagochilustaukumensis* Tzukerv. [*Lf*: Dwarf semishrub. *Lc*: Per. *D*: 18]

**Gen. 98. *Leonurus* L.**

340. *Leonurusincanus* V.I.Krecz. & Kuprian. [*Lf*: Herb. *Lc*: Per. *D*: 24]

**Gen. 99. *Salvia* L.**

341. *Salviatrautvetteri* Regel [*Lf*: Herb. *Lc*: Per. *D*: 28, 29]

**Gen. 100. *Scutellaria* L.**

342. *Scutellariaandrossovii* Juz. [*Lf*: Herb. *Lc*: Per. *D*: 15, 28]

343. *Scutellariakaratavica* Juz. [*Lf*: Herb. *Lc*: Per. *Cs*: RB. *D*: 28]

344. *Scutellariakurssanovii* Pavlov [*Lf*: Herb. *Lc*: Per. *D*: 28]

345. *Scutellarianavicularis* Juz. [*Lf*: Subshrub. *Lc*: Per. *Cs*: RB. *D*: 18, 24]

346. *Scutellariasubcaespitosa* Pavlov [*Lf*: Herb. *Lc*: Per. *Cs*: RB. *D*: 27, 28, 29]

347. *Scutellariatitovii* Juz. [*Lf*: Herb. *Lc*: Per. *D*: 26]

348. *Scutellariaturgaica* Juz. [*Lf*: Herb. *Lc*: Per. *D*: 9, 10, 10а, 11, 11а, 16]

**Gen. 101. *Thymus* L.**

349. *Thymuscrebrifolius* Klokov [*Lf*: Dwarf semishrub. *Lc*: Per. *D*: 10, 10а, 11а]

350. *Thymuseremita* Klokov [*Lf*: Dwarf semishrub. *Lc*: Per. *D*: 10а, 11]

351. *Thymuskaratavicus* Dmitrieva [*Lf*: Dwarf semishrub. *Lc*: Per. *D*: 28, 29]

352. *Thymusmagnificus* Klokov [*Lf*: Dwarf semishrub. *Lc*: Per. *D*: 25]

**Fam. 21. Liliaceae Juss.**

**Gen. 102. *Fritillaria* L.**

353. *Fritillariakolbintsevii* Rukšāns & Zubov [*Lf*: Herb. *Lc*: Per. *D*: 24]

**Gen. 103. *Gagea* Salisb.**

354. *Gageaalmaatensis* Levichev, A.Peterson & J.Peterson [*Lf*: Herb. *Lc*: Per. *D*: 25]

355. *Gageailiensis* Popov [*Lf*: Herb. *Lc*: Per. *D*: 16,18]

356. *Gageasarysuensis* Murz. [*Lf*: Herb. *Lc*: Per. *D*: 10, 11]

357. *Gageaularsaica* I.G.Levichev [*Lf*: Herb. *Lc*: Per. *D*: 28, 29]

**Gen. 104. *Tulipa* L.**

358. *Tulipaalberti* Regel [*Lf*: Herb. *Lc*: Per. *Cs*: RB. *D*: 10, 16, 18, 24, 26, 28, 29]

359. *Tulipaannae* J.de Groot & Zonn. [*Lf*: Herb. *Lc*: Per. *D*: 22, 24]

360. *Tulipaauliekolica* Perezhogin [*Lf*: Herb. *Lc*: Per. *D*: 2]

361. *Tulipaberkariensis* Rukšāns * [*Lf*: Herb. *Lc*: Per. *D*: 27, 28, 29]

362. *Tulipabrachystemon* Regel * [*Lf*: Herb. *Lc*: Per. *Cs*: RB. *D*: 24]

363. *Tulipadianae-verettiae* J.de Groot & Zonn. [*Lf*: Herb. *Lc*: Per. *D*: 22]

364. *Tulipaivasczenkoae* Epiktetov & Belyalov [*Lf*: Herb. *Lc*: Per. *D*: 24]

365. *Tulipakolbintsevii* Zonn. [*Lf*: Herb. *Lc*: Per. *D*: 24]

366. *Tulipalemmersii* Zonn., Peterse & J.de Groot [*Lf*: Herb. *Lc*: Per. *D*: 29]

367. *Tulipaorthopoda* Vved. * [*Lf*: Herb. *Lc*: Per. *D*: 28, 29]

368. *Tuliparegelii* Krasn. [*Lf*: Herb. *Lc*: Per. *Cs*: RB. *D*: 26]

369. *Tulipaturgaica* Perezhogin [*Lf*: Herb. *Lc*: Per. *D*: 9]

370. *Tulipasalsola* Rukšāns & Zubov [*Lf*: Herb. *Lc*: Per. *D*: 24]

**Fam. 22. Nitrariaceae Lindl.**

**Gen. 105. *Nitraria* L.**

371. *Nitrariailiensis* Banaev & Tomoshevich [*Lf*: Shrub. *Lc*: Per. *D*:18, 24]

**Gen. 106. *Tetradiclis* Steven ex M.Bieb.**

372. *Tetradicliscorniculata* Khalk. [*Lf*: Herb. *Lc*: An. *D*: 12]

**Fam. 23. Orobanchaceae Vent.**

**Gen. 107. *Euphrasia* L.**

373. *Euphrasiaintegriloba* J.J.Dmitriev & N.I.Rubtzov [*Lf*: Herb. *Lc*: An. *D*: 24]

374. *Euphrasiakarataviensis* Govor. [*Lf*: Herb. *Lc*: An. *D*: 28, 29]

**Gen. 108. *Pedicularis* L.**

375. *Pedicularisczuiliensis* Semiotr. [*Lf*: Herb. *Lc*: Per. *Cs*: RB. *D*: 26]

376. Pedicularisinterruptasubsp.tarbagataica (Semiotr.) Kamelin [*Lf*: Herb. *Lc*: Per. *Cs*: RB. *D*: 23]

377. *Pediculariskokpakensis* Semiotr. [*Lf*: Herb. *Lc*: Per. *D*: 25a]

378. *Pedicularismasalskyi* Semiotr. [*Lf*: Herb. *Lc*: Per. *D*: 29]

379. *Pedicularistransversa* Baimukhambetova [*Lf*: Herb. *Lc*: Per. *D*: 25а]

**Fam. 24. Plantaginaceae Juss.**

**Gen. 109. *Linaria* Mill.**

380. *Linariamacrophylla* Kuprian. [*Lf*: Herb. *Lc*: Per. *D*: 8]

**Fam. 25. Plumbaginaceae Juss.**

**Gen. 110. *Acantholimon* Boiss.**

380. *Acantholimonkaratavicum* Pavlov [*Lf*: Undershrub. *Lc*: Per. *D*: 17, 28]

382. *Acantholimonlinczevskii* Pavlov [*Lf*: Undershrub. *Lc*: Per. *Cs*: RB. *D*: 28]

383. *Acantholimonmikeschinii* Lincz. [*Lf*: Undershrub. *Lc*: Per. *D*: 28]

384. *Acantholimonminshelkense* Pavlov [*Lf*: Subshrub. *Lc*: Per. *D*: 28]

385. *Acantholimonpavlovii* Lincz. [*Lf*: Undershrub. *Lc*: Per. *D*: 29]

386. *Acantholimonsquarrosum* Pavlov [*Lf*: Undershrub. *Lc*: Per. *D*: 28]

**Gen. 111. *Limonium* Mill.**

387. *Limoniumbotschantzevii* (Lincz.) M.Malekm., Akhani & Borsch [*Lf*: Herb. *Lc*: Per. *D*: 21]

388. *Limoniummichelsonii* Lincz. [*Lf*: Herb. *Lc*: Per. *Cs*: RB. *D*: 24, 25, 25a]

**Fam. 26. Poaceae Barnhart**

**Gen. 112. *Agropyron* Gaertn**,

389. Agropyroncristatumsubsp.tarbagataicum (Plotn.) Tzvelev [*Lf*: Herb. *Lc*: Per. *D*: 22, 23]

**Gen. 113. *Elymus* L.**

390. *Elymusarcuatus* (Golosk.) Tzvelev [*Lf*: Herb. *Lc*: Per. *D*: 25]

391. *Elymusglaucissimus* (Popov) Tzvelev [*Lf*: Herb. *Lc*: Per. *D*: 25]

392. *Elymussibinicus* Kotukhov [*Lf*: Herb. *Lc*: Per. *D*: 22]

**Gen. 114. *Festuca* Tourn. ex L.**

393. *Festucairtyshensis* E.B.Alexeev [*Lf*: Herb. *Lc*: Per. *D*: 3]

394. *Festucasaurica* E.B.Alexeev [*Lf*: Herb. *Lc*: Per. *D*: 23]

**Gen. 115. *Leymus* Hochst.**

395. *Leymusdivaricatus* (Drobow) Tzvelev [*Lf*: Herb. *Lc*: Per. *D*: 25, 28]

**Gen. 116. *Limnas* Trin.**

396. *Limnasveresczaginii* Krylov & Schischk. [*Lf*: Herb. *Lc*: Per. *Cs*: RB. *D*: 22]

**Gen. 117. *Poa* L.**

397. *Poakoksuensis* Golosk. [*Lf*: Herb. *Lc*: Per. *D*: 24]

**Gen. 118. *Puccinellia* Parl.**

398. *Puccinelliamacropus* V.I.Krecz. [*Lf*: Herb. *Lc*: Per. *D*: 26]

**Gen. 119. *Stipa* L.**

399. *Stipaargillosa* Kotukhov * [*Lf*: Herb. *Lc*: Per. *D*: 22]

400. *Stipaaustroaltaica* Kotukhov [*Lf*: Herb. *Lc*: Per. *Cs*: RB. *D*: 22]

401. *Stipakarakabinica* Kotukhov [*Lf*: Herb. *Lc*: Per. *D*: 23]

402. *Stipakempirica* Kotukhov [*Lf*: Herb. *Lc*: Per. *D*: 23]

403. *Stipakotuchovii* M.Nobis [*Lf*: Herb. *Lc*: Per. *D*: 23]

**Fam. 27. Polygonaceae Juss.**

**Gen. 120. *Atraphaxis* L.**

404. *Atraphaxismuschketowii* Krasn. [*Lf*: Shrub. *Lc*: Per. *Cs*: RB. *D*: 25]

405. *Atraphaxisteretifolia* (Popov) Kom. [*Lf*: Shrub. *Lc*: Per. *Cs*: RB. *D*: 10, 11, 18]

**Gen. 121. *Calligonum* L.**

406. *Calligonumturbineum* Pavlov [Lf: Shrub. Lc: Per. *D*: 17, 18, 20]

**Gen. 122. *Rumex* L.**

407. *Rumexfischeri* Rchb. [*Lf*: Herb. *Lc*: Per. *D*: 24]

408. *Rumexkomarovii* Schischk. & Serg. [Lf: Herb. Lc: Per. *D*: 11]

**Fam. 28. Ranunculaceae Juss.**

**Gen. 123. *Aquilegia* L.**

409. *Aquilegiakaratavica* Mikeschin [*Lf*: Herb. *Lc*: Per. *Cs*: RB. *D*: 28]

410. *Aquilegiavitalii* Gamajun. [*Lf*: Herb. *Lc*: Per. *Cs*: RB. *D*: 24]

**Gen. 124. *Delphinium* L.**

411. *Delphiniumaustroaltaicum* A.L.Ebel [*Lf*: Herb. *Lc*: Per. *D*: 22]

412. *Delphiniumconnectens* Pachom. [*Lf*: Herb. *Lc*: Per. *D*: 25a]

413. *Delphiniumpavlovii* Kamelin [*Lf*: Herb. *Lc*: Per. *D*: 28]

**Gen. 125. *Ranunculus* L.**

414. *Ranunculuskarkaralensis* Schegol. [*Lf*: Herb. *Lc*: Per. *D*: 11a]

**Gen. 126. *Thalictrum* Tourn. ex L.**

415. *Thalictrumbykovii* Kotukhov * [*Lf*: Herb. *Lc*: Per. *D*: 22]

**Fam. 29. Rosaceae Juss.**

**Gen. 127. *Alchemilla* L.**

416. *Alchemillagoloskokovii* Juz. [*Lf*: Herb. *Lc*: Per. *D*: 24]

**Gen. 128. *Amelanchier* Medik.**

417. *Amelanchierturkestanica* Litv. [*Lf*: Shrub. *Lc*: Per. *D*: 11]

**Gen. 129. *Cotoneaster* Medik.**

418. *Cotoneasteralatavicus* Popov [*Lf*: Shrub. *Lc*: Per. *D*: 24, 25, 27, 29]

419. *Cotoneasteraltaicus* G.Klotz ex J.Fryer & B.Hylmö [*Lf*: Shrub. *Lc*: Per. *D*: 25]

420. *Cotoneasterkrasnovii* Pojark. [*Lf*: Shrub. *Lc*: Per. *D*: 18, 24, 25, 26]

421. *Cotoneasterneoantoninae* A.N.Vassiljeva [*Lf*: Shrub. *Lc*: Per. *D*: 24, 25]

422. *Cotoneasterpolyanthemus* E.L.Wolf [*Lf*: Shrub. *Lc*: Per. *D*: 24, 25]

423. *Cotoneastertalgaricus* Popov [*Lf*: Shrub. *Lc*: Per. *D*: 24, 25, 25а]

**Gen. 130. *Crataegus* L.**

424. Crataegusambiguasubsp.transcaspica (Pojark.) K.I.Chr. [*Lf*: Tree. *Lc*: Per. *D*: 13b]

**Gen. 131. *Potentilla* L.**

425. *Potentillakaratavica* Juz. [*Lf*: Herb. *Lc*: Per. *D*: 28]

426. *Potentillasalsa* Yu.A.Kotukhov [*Lf*: Herb. *Lc*: Per. *D*: 22]

427. *Potentillaschrenkiana* Regel [*Lf*: Herb. *Lc*: Per. *D*: 23, 24]

**Gen. 132. *Rosa* L.**

428. *Rosadsharkenti* Chrshan. [*Lf*: Shrub. *Lc*: Per. *D*: 18]

429. *Rosailiensis* Chrshan. [*Lf*: Shrub. *Lc*: Per. *D*: 17, 18]

430. *Rosapotentilliflora* Chrshan. & Popov [*Lf*: Shrub. *Lc*: Per. *D*: 25]

431. *Rosaschrenkiana* Crép. [*Lf*: Shrub. *Lc*: Per. *D*: 24]

**Gen. 133. *Spiraeanthus* (Fisch. & C.A.Mey.) Maxim.**

432. *Spiraeanthusschrenkianus* (Fisch. & C.A.Mey.) Maxim. [*Lf*: Shrub. *Lc*: Per. *Cs*: RB. *D*: 16, 21, 26, 28]

**Fam. 30. Rubiaceae Juss.**

**Gen. 134. *Galium* L.**

433. *Galiumkasachstanicum* Pachom. [*Lf*: Herb. *Lc*: Per. *D*: 25a]

434. *Galiumturgaicum* Knjaz. [*Lf*: Herb. *Lc*: Per. *D*: 7a]

435. *Galiumzaisanicum* Pinzhenina & Kupr. * [*Lf*: Herb. *Lc*: Per. *D*: 12]

**Gen. 135. *Rubia* L.**

436. *Rubiacretacea* Pojark. [*Lf*: Herb. *Lc*: Per. *Cs*: RB. *D*: 1, 6, 7a, 8, 13]

437. *Rubiapavlovii* Bajtenov & Myrz. [*Lf*: Herb. *Lc*: Per. *Cs*: RB. *D*: 28]

**Fam. 31. Rutaceae Juss.**

**Gen. 136. *Haplophyllum* A.Juss.**

438. *Haplophyllumeugenii-korovinii* Pavlov [*Lf*: Subshrub. *Lc*: Per. *Cs*: RB. *D*: 28]

439. *Haplophyllummulticaule* Vved. [*Lf*: Subshrub. *Lc*: Per. *D*: 5, 10, 14, 16, 18, 24, 26]

**Fam. 32. Scrophulariaceae Juss.**

**Gen. 137. *Scrophularia* Tourn. ex L.**

440. *Scrophulariadshungarica* Golosk. & Tzag. [*Lf*: Herb. *Lc*: Per. *Cs*: RB. *D*: 24]

441. *Scrophularianuraniae* Tzag. [*Lf*: Herb. *Lc*: Per. *Cs*: RB. *D*: 29]

**Fam. 33. Thymelaeaceae Juss.**

**Gen. 138. *Diarthron* Turcz.**

442. *Dendrostelleraammodendron* (Kar. & Kir.) Botsch. [*Lf*: Shrub. *Lc*: Per. *D*: 17, 18, 24, 25]

**Fam. 34. Zygophyllaceae R.Br.**

**Gen. 139. *Zygophyllum* L.**

443. *Zygophyllumbalchaschense* Boriss. [*Lf*: Herb. *Lc*: Per. *D*: 11, 16]

444. *Zygophyllumbetpakdalense* Golosk. & Semiotr. [*Lf*: Herb. *Lc*: Per. *D*: 16]

445. *Zygophyllumborissovae* Beier & Thulin [*Lf*: Herb. *Lc*: Per. *D*: 10, 11, 11а, 16]

446. *Zygophyllumfurcatum* C.A.Mey. [*Lf*: Herb. *Lc*: Per. *D*: 10, 11, 11а, 23, 24]

447. *Zygophyllumkaratavicum* Boriss. [*Lf*: Herb. *Lc*: Per. *Cs*: RB. *D*: 28]

448. *Zygophyllumkopalense* Boriss. [*Lf*: Herb. *Lc*: Per. *Cs*: RB. *D*: 16,18,25,26]

449. *Zygophyllumsteropterum* Schrenk [*Lf*: Herb. *Lc*: Per. *D*: 16, 17, 18, 26]

450. *Zygophyllumsubtrijugum* C.A.Mey. [*Lf*: Herb. *Lc*: Per. *D*: 3. 4. 10, 10а, 11, 16]

451. *Zygophyllumtaldykurganicum* Boriss. [*Lf*: Herb. *Lc*: Per. *D*: 18, 24]

**Notes***:

**Arthrophytumsubulifolium* Schrenk, according to POWO, is noted in Turkmenistan; however, according to our data and the scientific paper by Osmanali et al. (2019), this indication is incorrect. This species is a narrow-local endemic of the Chu-Ili Range in the south of Kazakhstan (Osmanali et al. 2019).

**Atriplexiljinii* Aellen, similar to *Arthrophytumsubulifolium* Schrenk, is erroneously reported in Turkmenistan, as per the POWO database. The distribution of *A.iljinii* is poorly studied, known mainly from type specimens. This species is observed in the northwest of the Mangistau Region, as well as in the Aktobe and Kyzylorda (Aralkum Desert) Regions of Kazakhstan (Suchorukow 2007).

**Ferulaleucographa* Korovin, according to POWO, is recorded in Uzbekistan; yet, according to the updated synopsis of *Apiaceae* of Kazakhstan and Central Asia (Pimenov 2020), the species grows only in Kazakhstan.

**Rinderaochroleuca* Kar. & Kir., as suggested by POWO, is observed in Altai in the Russian Federation; yet, our investigation did not find credible evidence supporting this information.

**Eutremahalophilum* (C.A.Mey.) Al-Shehbaz & Warwick was previously reported in China (Wu et al. 2008); however, German DA and Chen WL (2009) in their scientific paper did not confirm the presence of this species in China.

**Astragalusleucocalyx* Popov is recorded in POWO for Uzbekistan; nevertheless, Tojibaev et al. (2020b) in their scientific paper exclusively listed this species as occurring in Kazakhstan.

**Prangosmulticostata* Kljuykov & Lyskov., according to the scientific paper by Pimenov (2020), is a synonym for *Prangosdzhungarica* Pimenov. Further study is required to investigate whether species status of *Prangosmulticostata* is warranted.

**Centaureakryloviana* Serg. is not recognised in POWO and GBIF, probably due to nomenclature errors. However, according to the scientific paper by Kupriyanov (2018), this species is endemic to eastern Kazakhstan.

**Astragalusrariflorus* Ledeb., previously noted for Western Siberia (Krylov 1933), yet we did not find herbarium materials of this species from this territory. This species is also not recorded in the Flora of Siberia (Malyshev 1994).

**Lappulacristata* (Bunge) B. Fedtsch. in JBIF is recorded for Western Siberia, on the territory of the Russian Federation (Bochkov and Seregin 2022); however, the presence of this species lacks substantial verification. Notably, the Flora of Siberia (Malyshev 1997) does not include any records of this species. Therefore, further investigation is necessary to elucidate the occurrence of *Lappulacristata* within the territory of the Russian Federation.

**Onobrychisalatavica* Bajtenov was omitted from the list of flora of Kazakhstan (Abdulina 1999). Conspectus Florae Asiae Mediae (Kamelin et al. 1981) noted that this species needs to be re-collected to confirm species status.

**Tulipaberkariensis* Rukšāns in POWO is recognised as a synonym of *Tulipakaufmanniana* Regel., based on data from Everett (2013). Nevertheless, *T.berkariensis* from the Berkara Valley and other places in Kazakhstan has a lower amount of nuclear 2C DNA (based on the data flow cytometric measurement of nuclear DNA content) than authentic *T.kaufmanniana* from Uzbekistan (Zonneveld 2009). This discrepancy implies that *Tulipaberkariensis* should be recognised as a distinct taxonomic entity.

**Tulipaorthopoda* Vved. listed in POWO is recognised as a synonym for *Tulipabifloriformis* Vved., based on Christenhusz et al. (2013) and Everett (2013). This scientific paper also notes that the species status of *Tulipaorthopoda* should be warranted, based on differences in morphological characters and flowering period, but further fieldwork is required to establish the variability of *T.bifloriformis* in the wild.

**Tulipabrachystemon* Regel in POWO is recognised as a synonym for *Tulipatetraphylla* Regel, also according to Christenhusz et al. (2013) and Everett (2013). However, Zonneveld (2009) distinguishes *Tulipabrachystemon* Regel as an independent taxon, based on the nuclear DNA content (DNA value 2C).

**Galiumzaisanicum* Pinzhenina & Kupr. was described quite recently (Pinzhenina and Kupriyanov 2023), presumably due to its recent identification it is absent in the GBIF and POWO systems.

**Stipaargillosa* Kotukhov in POWO is erroneously listed for the Altai Republic of the Russian Federation. This species is described from the territory of East Kazakhstan. Type: Southern Altai, south-eastern foothills of the Azutau Ridge, Bulgartabaty tract, foothill desert, outcrops of tertiary clays, clay-rubbly areas, 05/22/1991, Yu. Kotukhov (LE) (Kotukhov 1998). The Azutau Ridge borders the basin of Lake Markakol from the south and is entirely situated within the territory of Kazakhstan (Yegorina et al. 2003).

**Thalictrumbykovii* Kotukhov, as well as the previous species in POWO, is erroneously listed for the Altai Republic of the Russian Federation. This species was also described from the territory of East Kazakhstan. Type: Southern Altai, eastern spurs of Azutau Ridge, Mramornaya Mount, Middle belt, 900–1100 m above sea level, south-eastern slope, steppe shrub meadows, 14/06/1984, Yu. Kotukhov (LE).

## Supplementary materials

Supplementary material 1 Checklist of subendemic taxa previously considered endemic in Kazakhstan Data type docx https://binary.pensoft.net/file/993673This dataset is made available under the Open Database License (http://opendatacommons.org/licenses/odbl/1.0/). The Open Database License (ODbL) is a license agreement intended to allow users to freely share, modify, and use this Dataset while maintaining this same freedom for others, provided that the original source and author(s) are credited.Serik A. Kubentayev, Daniyar T. Alibekov, Yuri V. Perezhogin, Georgy A. Lazkov, Andrey N. Kupriyanov, Alexander L. Ebel, Klara S. Izbastina, Olga V. Borodulina, Balsulu B. Kubentayeva

Supplementary material 2 Former endemics of Kazakhstan that are now reclassified as synonyms for species exhibiting broader geographical distributions Data type docx https://binary.pensoft.net/file/993674This dataset is made available under the Open Database License (http://opendatacommons.org/licenses/odbl/1.0/). The Open Database License (ODbL) is a license agreement intended to allow users to freely share, modify, and use this Dataset while maintaining this same freedom for others, provided that the original source and author(s) are credited.Serik A. Kubentayev, Daniyar T. Alibekov, Yuri V. Perezhogin, Georgy A. Lazkov, Andrey N. Kupriyanov, Alexander L. Ebel, Klara S. Izbastina, Olga V. Borodulina, Balsulu B. Kubentayeva
