# Supplementary material for: ﻿Revised checklist of endemic vascular plants of Kazakhstan
Source: PhytoKeys. 2024 Feb 28;238:241–79. doi: 10.3897/phytokeys.238.114475 (PMC10918586; doi:10.3897/phytokeys.238.114475)
Supplement: Supplementary material 1 — Checklist of subendemic taxa previously considered endemic in Kazakhstan [file phytokeys-238-241_article-114475__-s001.docx]

Supplementary data 1

**Checklist of subendemic taxa previously considered endemic in Kazakhstan**

| № | **Species name** | **Family** | **Recorded in adjacent countries and replaced synonym in this study** | **Reference** |
| --- | --- | --- | --- | --- |
|  | *Anabasis jaxartica* (Bunge) Benth. ex Iljin | Amaranthaceae Juss. | Iran, Kyrgyzstan, Tadzhikistan, Turkmenistan, Uzbekistan | POWO |
|  | *Arthrophytum iliense* Iljin | Amaranthaceae Juss. | China | Wu et al. 2008 |
|  | *Arthrophytum korovinii* Botsch. | Amaranthaceae Juss. | China | Wu et al. 2008 |
|  | *Atriplex crassifolia* C.A.Mey. | Amaranthaceae Juss. | Russia | POWO |
|  | *Atriplex pungens* Trautv. | Amaranthaceae Juss. | Turkey, Turkmenistan | POWO |
|  | *Climacoptera kasakorum* (Iljin) Botsch. | Amaranthaceae Juss. | Uzbekistan | Hassler (1994 - 2024) |
|  | *Corispermum laxiflorum* Schrenk | Amaranthaceae Juss. | Uzbekistan | POWO |
|  | *Kalidium schrenkianum* Bunge ex Ung.-Sternb. | Amaranthaceae Juss. | China, Kyrgyzstan, Tadzhikistan | POWO |
|  | *Rhaphidophyton regelii* (Bunge) Iljin | Amaranthaceae Juss. | Kyrgyzstan | Lazkov and Sultanova 2011 |
|  | *Salsola halimocnemis* Botsch. | Amaranthaceae Juss. | Uzbekistan | Osmonali 2019 |
|  | *Suaeda kossinskyi* Iljin | Amaranthaceae Juss. | China, Mongolia, Russia | Hassler (1994 - 2024) |
|  | *Allium drobovii* Vved. | Amaryllidaceae J.St.-Hil. | Uzbekistan | POWO |
|  | *Allium inops* Vved. | Amaryllidaceae J.St.-Hil. | Kyrgyzstan | Lazkov and Sennikov 2017a |
|  | *Allium kurssanovii*Popov | Amaryllidaceae J.St.-Hil. | China, Kyrgyzstan | POWO; Wu et al. 2008 |
|  | *Allium lutescens* Vved. | Amaryllidaceae J.St.-Hil. | Kyrgyzstan, Uzbekistan | POWO |
|  | *Allium margaritae* B.Fedtsch. | Amaryllidaceae J.St.-Hil. | Kyrgyzstan | POWO |
|  | *Allium petraeum* Kar. & Kir. | Amaryllidaceae J.St.-Hil. | China, Kyrgyzstan | Lazkov and Sultanova 2014 |
|  | *Allium pskemense* B.Fedtsch. | Amaryllidaceae J.St.-Hil. | Kyrgyzstan, Uzbekistan | POWO |
|  | *Allium robustum* Kar. & Kir. | Amaryllidaceae J.St.-Hil. | China | Friesen et al. 2021a |
|  | *Allium talassicum* Regel | Amaryllidaceae J.St.-Hil. | Kyrgyzstan, Tadzhikistan, Uzbekistan | POWO |
|  | *Allium tekesicola* Regel | Amaryllidaceae J.St.-Hil. | China | Wu et al. 2008 |
|  | *Allium trachyscordum* Vved. | Amaryllidaceae J.St.-Hil. | Kyrgyzstan | POWO |
|  | *Allium tschulaktavicum*Bajtenov & Nelina | Amaryllidaceae J.St.-Hil. | China | Hassler (1994 - 2024) |
|  | *Allium valentinae* Pavlov | Amaryllidacea | Kyrgyzstan | Sennikov and Lazkov 2023 |
|  | *Allium vvedenskyanum* Pavlov | Amaryllidaceae J.St.-Hil. | Kyrgyzstan | POWO |
|  | *Aulacospermum tianschanicum* (Korovin) C.Norman | Apiaceae Lindl. | Kyrgyzstan, Uzbekistan | Pimenov 2020 |
|  | *Elwendia vaginata* (Korovin) Pimenov & Kljuykov | Apiaceae Lindl. | Uzbekistan | POWO |
|  | *Ferula ceratophylla* Regel & Schmalh. | Apiaceae Lindl. | Uzbekistan | Pimenov 2020 |
|  | *Ferula iliensis* Krasn. ex Korovin | Apiaceae Lindl. | China | Pimenov 2017 |
|  | *Ferula leiophylla* Korovin | Apiaceae Lindl. | China, Kyrgyzstan | Pimenov 2020 |
|  | *Ferula potaninii* Korovin ex Pavlov | Apiaceae Lindl. | China, Mongolia | Pimenov 2020 |
|  | *Ledebouriella multiflora* (Ledeb.) H. Wolff | Apiaceae Lindl. | China | Hassler (1994 - 2024); Pimenov 2017 |
|  | *Palimbia defoliata* (Ledeb.) Korovin | Apiaceae Lindl. | China | Hassler (1994 - 2024); Pimenov 2017 |
|  | *Pastinacopsis glacialis* Golosk. | Apiaceae Lindl. | China, Kyrgyzstan | POWO |
|  | *Prangos herderi* (Regel) Herrnst. & Heyn | Apiaceae Lindl. | China | Hassler (1994 - 2024);Pimenov 2020 |
|  | *Prangos ledebourii* Herrnst. & Heyn | Apiaceae Lindl. | China, Mongolia | Pimenov 2017 |
|  | *Semenovia rubtzovii* (Schischk.) Manden. | Apiaceae Lindl. | China | Pimenov 2017 |
|  | *Seseli abolinii* (Korovin) Schischk. | Apiaceae Lindl. | China, Mongolia | Pimenov 2020 |
|  | *Seseli asperulum* (Trautv.) Schischk. | Apiaceae Lindl. | China | Pimenov 2017 |
|  | *Seseli eriocarpum* (Schrenk) B.Fedtsch. | Apiaceae Lindl. | China, Mongolia | Pimenov 2017 |
|  | *Seseli eriocephalum* (Pall. ex Spreng.) Schischk. | Apiaceae Lindl. | China, Russia, Turkmenistan, Uzbekistan | POWO |
|  | *Seseli coronatum* Ledeb. | Apiaceae Lindl. | China | Pimenov 2017 |
|  | *Seseli incanum* (Stephan ex Willd.) B.Fedtsch. | Apiaceae Lindl. | China | Pimenov 2017 |
|  | *Seseli setiferum* Pimenov & Sdobnina | Apiaceae Lindl. | Uzbekistan | Pimenov 2020 |
|  | *Sium medium* Fisch. & C.A.Mey. | Apiaceae Lindl. | Afghanistan, China, India, Kyrgyzstan, Pakistan, Tadzhikistan, Uzbekistan | Hassler (1994 - 2024); Pimenov, 2020 |
|  | *Apocynum pictum* Schrenk | Apocynaceae Juss. | China, Kyrgyzstan, Mongolia, Tadzhikistan | POWO |
|  | *Achillea karatavica* Kamelin | Asteraceae Bercht. & J.Presl | Kyrgyzstan, Uzbekistan | Kupriyanov and Kulemin 2023 |
|  | *Achillea kamelinii* Kupr. | Asteraceae Bercht. & J.Presl | Tadzhikistan, Uzbekistan | GBIF |
|  | *Achillea tianschanica* Kupr. & Kulemin | Asteraceae Bercht. & J.Presl | Kyrgyzstan | Kupriyanov and Kulemin 2023 |
|  | *Alfredia fetissowii* Iljin | Asteraceae Bercht. & J.Presl | China | POWO |
|  | *Arctium triflorum* Kuntze | Asteraceae Bercht. & J.Presl | Iran, Kyrgyzstan, Tadzhikistan, Turkmenistan, Uzbekistan | POWO |
|  | *Arctium vavilovii* (Kult.) S.López, Romasch., Susanna & N.Garcia | Asteraceae Bercht. & J.Presl | Kyrgyzstan | POWO |
|  | *Artemisia amoena* Poljakov | Asteraceae Bercht. & J.Presl | Mongolia, China | POWO |
|  | *Artemisia cina* O.Berg | Asteraceae Bercht. & J.Presl | Iran, Uzbekistan | POWO |
|  | *Artemisia halophila* Krasch. | Asteraceae Bercht. & J.Presl | Turkmenistan, Uzbekistan | POWO |
|  | *Artemisia heptapotamica* Poljakov | Asteraceae Bercht. & J.Presl | China, Kyrgyzstan, Mongolia | POWO |
|  | *Artemisia karatavica* Krasch. & Abolin ex Poljakov | Asteraceae Bercht. & J.Presl | China, Kyrgyzstan | POWO |
|  | *Artemisia mucronulata* Poljakov | Asteraceae Bercht. & J.Presl | Kyrgyzstan | POWO |
|  | *Artemisia saissanica* (Krasch.) Filatova | Asteraceae Bercht. & J.Presl | Mongolia | POWO |
|  | *Artemisia scopiformis* Ledeb. | Asteraceae Bercht. & J.Presl | China | POWO |
|  | *Artemisia semiarida* (Krasch. & Lavrenko) Filatova | Asteraceae Bercht. & J.Presl | China | POWO |
|  | *Artemisia succulenta* Ledeb. | Asteraceae Bercht. & J.Presl | China, Mongolia, Turkmenistan, Uzbekistan, | POWO |
|  | *Artemisia tomentella* Trautv. | Asteraceae Bercht. & J.Presl | Mongolia, Russia | POWO |
|  | *Artemisia transiliensis* Poljakov | Asteraceae Bercht. & J.Presl | China, Kyrgyzstan | POWO |
|  | *Chondrilla kusnezovii* Iljin | Asteraceae Bercht. & J.Presl | Kyrgyzstan | POWO |
|  | *Cousinia baranovii* Tscherneva | Asteraceae Bercht. & J.Presl | Kyrgyzstan | POWO |
|  | *Cousinia chrysantha* Kult. | Asteraceae Bercht. & J.Presl | Uzbekistan | POWO |
|  | *Cousinia kazachorum* Juz. ex Tscherneva | Asteraceae Bercht. & J.Presl | Kyrgyzstan | POWO |
|  | *Cousinia minkwitziae* Bornm. | Asteraceae Bercht. & J.Presl | Kyrgyzstan | POWO |
|  | *Cousinia mollis* Schrenk | Asteraceae Bercht. & J.Presl | Afghanistan, Kyrgyzstan, Tadzhikistan, Turkmenistan, Uzbekistan | POWO |
|  | *Cousinia regelii* C.Winkl. | Asteraceae Bercht. & J.Presl | Kyrgyzstan, Tadzhikistan, Uzbekistan | POWO |
|  | *Cousinia talassica* (Kult.) Juz. ex Tschern. | Asteraceae Bercht. & J.Presl | Kyrgyzstan | POWO |
|  | *Cousinia tianschanica* Kult. | Asteraceae Bercht. & J.Presl | Kyrgyzstan, Tadzhikistan, Uzbekistan | POWO |
|  | *Cousinia transiliensis* Juz. | Asteraceae Bercht. & J.Presl | Kyrgyzstan | POWO |
|  | *Echinops albicaulis* Kar. & Kir. | Asteraceae Bercht. & J.Presl | China | GBIF |
|  | *Echinops saissanicus* (B.Keller) Bobrov | Asteraceae Bercht. & J.Presl | Russia | POWO |
|  | *Echinops talassicus* Golosk. | Asteraceae Bercht. & J.Presl | China, Kyrgyzstan | Wu et al. 2008 |
|  | *Erigeron violaceus* Popov | Asteraceae Bercht. & J.Presl | Kyrgyzstan | POWO |
|  | *Galatella regelii* Tzvelev | Asteraceae Bercht. & J.Presl | China | Wu et al. 2008 |
|  | *Galatella tianschanica* Novopokr. | Asteraceae Bercht. & J.Presl | China | Wu et al. 2008 |
|  | *Jurinea adenocarpa* Schrenk | Asteraceae Bercht. & J.Presl | China | Wu et al. 2008 |
|  | *Jurinea dshungarica* (Rubtzov) Iljin | Asteraceae Bercht. & J.Presl | China | Kupriyanov 2018 |
|  | *Jurinea kapelkini* O.Fedtsch. | Asteraceae Bercht. & J.Presl | Russia | Kupriyanov 2018 |
|  | *Jurinea lipskyi* Iljin | Asteraceae Bercht. & J.Presl | China | Wu et al. 2008 |
|  | *Jurinea margalensis* Iljin | Asteraceae Bercht. & J.Presl | Mongolia | Baasanmunkh et al. 2022 |
|  | *Jurinea mugodsharica* Iljin | Asteraceae Bercht. & J.Presl | Russia | POWO |
|  | *Jurinea tenuiloba* Bunge | Asteraceae Bercht. & J.Presl | Russia, Ukraine | Kupriyanov 2018 |
|  | *Klasea dissecta* (Ledeb.) L.Martins | Asteraceae Bercht. & J.Presl | China | Wu et al. 2008 |
|  | *Klasea suffruticulosa* (Schrenk) L.Martins | Asteraceae Bercht. & J.Presl | China, Kyrgyzstan | Wu et al. 2008 |
|  | *Rhaponticum nitidum* Fisch. ex DC. | Asteraceae Bercht. & J.Presl | Uzbekistan | POWO |
|  | *Microcephala subglobosa* (Krasch.) Pobed. | Asteraceae Bercht. & J.Presl | China, Kyrgyzstan, Tadzhikistan, Turkmenistan | POWO |
|  | *Phalacrachena calva* (Ledeb.) Iljin | Asteraceae Bercht. & J.Presl | Mongolia | Baasanmunkh et al. 2022 |
|  | *Pilosella kumbelica* (B.Fedtsch. & Nevski) Sennikov | Asteraceae Bercht. & J.Presl | Kyrgyzstan | POWO |
|  | *Prenanthes mira* (Pavlov) Kamelin | Asteraceae Bercht. & J.Presl | Kyrgyzstan | Tojibaev et al. 2020a |
|  | *Psephellus turgaicus* (Klokov) A.L.Ebel | Asteraceae Bercht. & J.Presl | Russia | POWO |
|  | *Richteria semenovii* (Herder) Sonboli & Oberpr. | Asteraceae Bercht. & J.Presl | Tadzhikistan | Nowak et al. 2020 |
|  | *Saussurea blanda* Schrenk | Asteraceae Bercht. & J.Presl | China | Wu et al. 2008 |
|  | *Saussurea caespitans* Iljin | Asteraceae Bercht. & J.Presl | Kyrgyzstan | POWO |
|  | *Saussurea coronata* Schrenk. | Asteraceae Bercht. & J.Presl | China, Mongolia | Wu et al. 2008; Baasanmunkh et al. 2022 |
|  | *Saussurea lipschitzii* Filatova | Asteraceae Bercht. & J.Presl | Mongolia | Baasanmunkh et al. 2022 |
|  | *Saussurea robusta* Ledeb. | Asteraceae Bercht. & J.Presl | China, Kyrgyzstan, Russia | POWO |
|  | *Serratula kirghisorum* Iljin | Asteraceae Bercht. & J.Presl | Mongolia, Russia | POWO |
|  | *Tanacetum scopulorum* (Krasch.) Tzvelev | Asteraceae Bercht. & J.Presl | China | Wu et al. 2008 |
|  | *Taraxacum alatavicum* Schischk. | Asteraceae Bercht. & J.Presl | Afghanistan, Iran | POWO |
|  | *Taraxacum calcareum* Korol. | Asteraceae Bercht. & J.Presl | Kyrgyzstan | POWO |
|  | *Taraxacum glabellum* Schischk. | Asteraceae Bercht. & J.Presl | Kyrgyzstan, Turkmenistan | POWO |
|  | *Taraxacum goloskokovii* Schischk. | Asteraceae Bercht. & J.Presl | China, Kyrgyzstan, Mongolia, Tadzhikistan | POWO |
|  | *Taraxacum longipyramidatum* Schischk. | Asteraceae Bercht. & J.Presl | Kyrgyzstan, Tadzhikistan | POWO |
|  | *Taraxacum majus* Schischk. | Asteraceae Bercht. & J.Presl | Kyrgyzstan, Tadzhikistan | POWO |
|  | *Taraxacum microspermum* Schischk. | Asteraceae Bercht. & J.Presl | Kyrgyzstan, Mongolia, Russia | POWO |
|  | *Taraxacum pavlovii* Orazova | Asteraceae Bercht. & J.Presl | Kyrgyzstan | POWO |
|  | *Taraxacum pingue* Schischk. | Asteraceae Bercht. & J.Presl | China, Kyrgyzstan, Mongolia, Tadzhikistan, Russia | POWO |
|  | *Taraxacum pseudoatratum* Orazova | Asteraceae Bercht. & J.Presl | China, Kyrgyzstan, Mongolia, Russia | POWO |
|  | *Taraxacum schischkinii* Korol. | Asteraceae Bercht. & J.Presl | Kyrgyzstan | POWO |
|  | *Taraxacum songoricum* Schischk. | Asteraceae Bercht. & J.Presl | Mongolia | POWO |
|  | *Taraxacum subglaciale* Schischk. | Asteraceae Bercht. & J.Presl | China, Kyrgyzstan, Tadzhikistan | POWO |
|  | *Taraxacum tujuksuense* Orazova | Asteraceae Bercht. & J.Presl | Kyrgyzstan | POWO |
|  | *Taraxacum turgaicum* Schischk. | Asteraceae Bercht. & J.Presl | Russia, Mongolia | POWO |
|  | *Taraxacum vitalii* Orazova | Asteraceae Bercht. & J.Presl | Kyrgyzstan | POWO |
|  | *Tephroseris pyroglossa* (Kar. & Kir.) Holub | Asteraceae Bercht. & J.Presl | China | Ma and Xu 2019 |
|  | *Tragopogon dubjanskyi* Krasch. & Nikitin | Asteraceae Bercht. & J.Presl | Russia | Mavrodiev 2005 |
|  | *Tragopogon scoparius* S.A.Nikitin | Asteraceae Bercht. & J.Presl | Russia | POWO |
|  | *Trichanthemis radiata* Krasch. & Vved. | Asteraceae Bercht. & J.Presl | Kyrgyzstan | POWO |
|  | *Betula psammophila* V.N.Vassil. | Betulaceae Gray | Russia | POWO |
|  | *Craniospermum subfloccosum* Krylov | Boraginaceae Juss. | China, Mongolia, Russia | POWO |
|  | *Lappula brachycentroides* Popov | Boraginaceae Juss. | Mongolia, Russia | POWO |
|  | *Lappula dzharkentica* (Popov) Popov | Boraginaceae Juss. | Russia | POWO |
|  | *Lappula duplicicarpa* Pavlov | Boraginaceae Juss. | China, Mongolia | POWO |
|  | *Lappula lipskyi* Popov | Boraginaceae Juss. | China, Mongolia | POWO |
|  | *Lappula macra* Popov ex Pavlov | Boraginaceae Juss. | China | Hassler (1994 - 2024) |
|  | *Lappula macrantha* (Ledeb.) Gürke | Boraginaceae Juss. | China, Mongolia | POWO |
|  | *Lappula rupestris* (Schrenk) Gürke | Boraginaceae Juss. | Kyrgyzstan | POWO |
|  | *Mertensia tarbagataica* B.Fedtsch. | Boraginaceae Juss. | China, Mongolia | POWO |
|  | *Rindera echinata* Regel | Boraginaceae Juss. | Uzbekistan | Ovchinnikova 2021 |
|  | *Erysimum croceum* Popov | Brassicaceae Burnett | China, Kyrgyzstan | POWO, Lazkov and Sultanova 2014 |
|  | *Erysimum kamelinii* D.A.German | Brassicaceae Burnett | Kyrgyzstan | GBIF |
|  | *Erysimum kotuchovii* D.A.German | Brassicaceae Burnett | China, Mongolia | German 2005;  Kiefer et al. 2014 |
|  | *Erysimum transiliense* Popov | Brassicaceae Burnett | China, Kyrgyzstan, Mongolia, Russia | German 2006a |
|  | *Eutrema pseudocordifolium* Popov | Brassicaceae Burnett | Kyrgyzstan | POWO |
|  | *Farsetia spathulata* Kar. & Kir. | Brassicaceae Burnett | China | German et al. 2012 |
|  | *Leiospora beketovii* (Krasn.) D.A.German & Al-Shehbaz | Brassicaceae Burnett | China, Kyrgyzstan | POWO |
|  | *Lepidium brachyotum* (Kar. & Kir.) Al-Shehbaz | Brassicaceae Burnett | China | Zhou 2001 |
|  | *Lepidium cardiophyllum* (Pavlov) Al-Shehbaz | Brassicaceae Burnett | Uzbekistan | German et al. 2013 |
|  | *Neotorularia brevipes* (Kar. & Kir.) Hedge & J.Léonard | Brassicaceae Burnett | Afghanistan, China, Kyrgyzstan, Mongolia, Pakistan, Turkmenistan | POWO |
|  | *Odontarrhena fedtschenkoana* (N. Busch) D. A. German | Brassicaceae Burnett | China | German and Chen 2009 |
|  | *Odontarrhena szarabiaca* (Nyár.) D.A.German | Brassicaceae Burnett | China | German and Chen 2009 |
|  | *Parrya albida* Popov | Brassicaceae Burnett | Kyrgyzstan, Tadzhikistan, Uzbekistan | POWO |
|  | *Parrya asperrima* (B.Fedtsch.) Popov | Brassicaceae Burnett | Kyrgyzstan, Tadzhikistan, Uzbekistan | POWO |
|  | *Parrya australis* Pavlov | Brassicaceae Burnett | Kyrgyzstan | German 2013 |
|  | *Parrya lancifolia* Popov | Brassicaceae Burnett | China, Kyrgyzstan | POWO |
|  | *Rhammatophyllum erysimoides* (Kar. & Kir.) Al-Shehbaz & O.Appel | Brassicaceae Burnett | Mongolia | German et al. 2006; Baasanmunkh et al. 2022 |
|  | *Sisymbrium subspinescens* Bunge | Brassicaceae Burnett | China, Kyrgyzstan, Mongolia, Tadzhikistan, Uzbekistan | POWO; German and Chen 2009 |
|  | *Eremogone asiatica* (Schischk.) Ikonn. | Caryophyllaceae Juss. | Mongolia | Baasanmunkh et al. 2022; Hassler (1994 - 2024) |
|  | *Eremogone potaninii* (Schischk.) Rabeler & W.L.Wagner | Caryophyllaceae Juss. | China | POWO |
|  | *Gypsophila krascheninnikovii* Schischk. | Caryophyllaceae Juss. | Turkmenistan | POWO |
|  | *Gypsophila rupestris* Kupr. | Caryophyllaceae Juss. | Russia | POWO |
|  | *Gypsophila spathulifolia* (Fisch. & C.A.Mey.) Fenzl | Caryophyllaceae Juss. | Kyrgyzstan, Turkmenistan, Uzbekistan | POWO |
|  | *Silene korshinskyi* Schischk. | Caryophyllaceae Juss. | Kyrgyzstan, Tadzhikistan | POWO |
|  | *Silene pugionifolia* Popov | Caryophyllaceae Juss. | Kyrgyzstan, Uzbekistan | POWO |
|  | *Convolvulus subsericeus* Schrenk ex Fisch. & C.A.Mey. | Convolvulaceae Juss. | China | Ma and Xu 2019 |
|  | *Rosularia turkestanica* (Regel & C.Winkl.) A.Berger | Crassulaceae J.St.-Hil. | China, Kyrgyzstan | POWO |
|  | *Carex minutiscabra* Kük. ex V.I.Krecz. | Cyperaceae Juss. | China | Grubov 1967 |
|  | *Schoenoplectus litoralis* subsp. *kasachstanicus* (Dobrochot.) Soják | Cyperaceae Juss. | Uzbekistan | POWO |
|  | *Ephedra lomatolepis* Schrenk | Ephedraceae Dumort. | China, Russia, Mongolia | POWO |
|  | *Euphorbia × popovii* Rotschild | Euphorbiaceae Juss. | Turkmenistan, Uzbekistan | POWO |
|  | *Euphorbia andrachnoides* Schrenk | Euphorbiaceae Juss. | China, Russia, Mongolia | POWO |
|  | *Euphorbia blepharophylla* C.A.Mey. | Euphorbiaceae Juss. | China, Mongolia | POWO |
|  | *Euphorbia irgisensis* Litv. | Euphorbiaceae Juss. | Turkmenistan | POWO |
|  | *Euphorbia macrorhiza* C.A.Mey. | Euphorbiaceae Juss. | China, Russia, Mongolia | POWO |
|  | *Euphorbia microcarpa* (Prokh.) Krylov | Euphorbiaceae Juss. | China, Russia | POWO |
|  | *Euphorbia sewerzowii* (Prokh.) Pavlov | Euphorbiaceae Juss. | Kyrgyzstan, Uzbekistan | Hassler (1994 - 2024) |
|  | *Euphorbia sororia* Schrenk | Euphorbiaceae Juss. | China, Iran, Kyrgyzstan, Pakistan, Russia, Tadzhikistan | POWO |
|  | *Astragalus aktiubensis* Sytin | Fabaceae Lindl. | Russia | POWO |
|  | *Astragalus alabugensis* B.Fedtsch. | Fabaceae Lindl. | Kyrgyzstan, Uzbekistan | POWO |
|  | *Astragalus albicans* Bong. | Fabaceae Lindl. | China, Mongolia | POWO |
|  | *Astragalus amabilis* Popov | Fabaceae Lindl. | China | POWO |
|  | *Astragalus aschuturi* B.Fedtsch. | Fabaceae Lindl. | Kyrgyzstan, Tadzhikistan, Uzbekistan | POWO |
|  | *Astragalus aulieatensis* Popov | Fabaceae Lindl. | Kyrgyzstan | POWO |
|  | *Astragalus austrodshungaricus* Golosk. | Fabaceae Lindl. | China | POWO |
|  | *Astragalus badamensis* Popov | Fabaceae Lindl. | Kyrgyzstan | GBIF |
|  | *Astragalus brachypus* Schrenk | Fabaceae Lindl. | China, Kyrgyzstan | POWO |
|  | *Astragalus chaetodon* Bunge | Fabaceae Lindl. | Uzbekistan | POWO |
|  | *Astragalus chlorodontus* Bunge | Fabaceae Lindl. | Kyrgyzstan | POWO |
|  | *Astragalus compositus* Pavlov | Fabaceae Lindl. | Kyrgyzstan, Pakistan, Uzbekistan | POWO |
|  | *Astragalus consanguineus* Bong. & C.A.Mey. | Fabaceae Lindl. | China, Russia, Mongolia | POWO |
|  | *Astragalus cysticalyx* Ledeb. | Fabaceae Lindl. | China | POWO |
|  | *Astragalus dsharkenticus* Popov | Fabaceae Lindl. | China, Kyrgyzstan | POWO |
|  | *Astragalus glomeratus* Ledeb. | Fabaceae Lindl. | Russia, Mongolia | POWO |
|  | *Astragalus gontscharovii* Vassilcz. | Fabaceae Lindl. | China, Kyrgyzstan, Uzbekistan | POWO |
|  | *Astragalus kasachstanicus* Golosk. | Fabaceae Lindl. | China, Mongolia | POWO |
|  | *Astragalus kendyrlykii* Popov | Fabaceae Lindl. | China | Yakovlev 2003 |
|  | *Astragalus kurtschumensis* Bunge | Fabaceae Lindl. | China, Mongolia | POWO |
|  | *Astragalus kustanaicus* Popov | Fabaceae Lindl. | Russia | Knyazev 2016 |
|  | *Astragalus macropetalus* Schrenk | Fabaceae Lindl. | Uzbekistan | Tojibaev et al. 2020b |
|  | *Astragalus melanocladus* Lipsky | Fabaceae Lindl. | China | Ma and Xu 2019 |
|  | *Astragalus mugosaricus* Bunge | Fabaceae Lindl. | Russia | Riabinina and Kniazev 2009 |
|  | *Astragalus nicolaii* Boriss. | Fabaceae Lindl. | China | Ho and Fu 1993 |
|  | *Astragalus ninae* Pavlov | Fabaceae Lindl. | Uzbekistan | POWO |
|  | *Astragalus ornithorrhynchus* Popov | Fabaceae Lindl. | China | POWO |
|  | *Astragalus pavlovianus* Gamajun. | Fabaceae Lindl. | China | Roskov et al. 2006 |
|  | *Astragalus polyceras* Kar. & Kir. | Fabaceae Lindl. | China | Wu et al. 2008 |
|  | *Astragalus rupifragiformis* Popov | Fabaceae Lindl. | Kyrgyzstan | POWO |
|  | *Astragalus saccocalyx* Schrenk | Fabaceae Lindl. | China | POWO |
|  | *Astragalus salsugineus* Kar. & Kir. | Fabaceae Lindl. | Turkmenistan | POWO |
|  | *Astragalus sogotensis* Lipsky | Fabaceae Lindl. | China | POWO |
|  | *Astragalus sphaerophysa* Kar. & Kir. | Fabaceae Lindl. | China | POWO |
|  | *Astragalus subarcuatus* Popov | Fabaceae Lindl. | Russia, Uzbekistan | POWO |
|  | *Astragalus temirensis* Popov | Fabaceae Lindl. | Russia | POWO |
|  | *Astragalus trichanthus*Golosk. | Fabaceae Lindl. | Kyrgyzstan | POWO |
|  | *Astragalus unijugus* Bunge | Fabaceae Lindl. | China | POWO |
|  | *Astragalus ustiurtensis* Bunge | Fabaceae Lindl. | Turkmenistan | POWO |
|  | *Caragana balchaschensis* (Kasn. ex Kom.) Pojark. | Fabaceae Lindl. | Russia | Hassler (1994 - 2024) |
|  | *Caragana tragacanthoides* (Pall.) Poir. | Fabaceae Lindl. | China | Hassler (1994 - 2024) |
|  | *Chesneya dshungarica* Golosk. | Fabaceae Lindl. | Kyrgyzstan | POWO |
|  | *Eremosparton songoricum* (Litv.) Vassilcz. | Fabaceae Lindl. | China, Kyrgyzstan | POWO |
|  | *Hedysarum aculeatum* Golosk. | Fabaceae Lindl. | Kyrgyzstan | POWO |
|  | *Hedysarum acutifolium* Bajtenov | Fabaceae Lindl. | Kyrgyzstan | POWO |
|  | *Hedysarum dmitrievae* Bajtenov | Fabaceae Lindl. | Kyrgyzstan, Uzbekistan | POWO |
|  | *Hedysarum krylovii* Sumnev. | Fabaceae Lindl. | China, Mongolia | POWO |
|  | *Hedysarum linczevskyi* Bajtenov | Fabaceae Lindl. | Mongolia | Baasanmunkh et al. 2022 |
|  | *Hedysarum subglabrum* (Kar. & Kir.) B.Fedtsch. | Fabaceae Lindl. | Kyrgyzstan | POWO |
|  | *Oxytropis arystangalievii*Bajtenov | Fabaceae Lindl. | Kyrgyzstan | POWO |
|  | *Oxytropis avis* Saposhn. | Fabaceae Lindl. | China | Roskov et al. 2006 |
|  | *Oxytropis caespitosula* Gontsch. | Fabaceae Lindl. | Kyrgyzstan, Uzbekistan | POWO |
|  | *Oxytropis cana* Bunge | Fabaceae Lindl. | China, Kyrgyzstan | POWO |
|  | *Oxytropis chionophylla* Schrenk | Fabaceae Lindl. | Mongolia | POWO |
|  | *Oxytropis chorgossica* Vassilcz. | Fabaceae Lindl. | China | Roskov et al. 2006 |
|  | *Oxytropis cuspidata* Bunge | Fabaceae Lindl. | China | Roskov et al. 2006 |
|  | *Oxytropis fetisowii* Bunge | Fabaceae Lindl. | China | Roskov et al. 2006 |
|  | *Oxytropis hystrix* Schrenk | Fabaceae Lindl. | China | POWO |
|  | *Oxytropis ketmenica* Saposhn. | Fabaceae Lindl. | China | Roskov et al. 2006 |
|  | *Oxytropis ornata* Vassilcz. | Fabaceae Lindl. | Uzbekistan | POWO |
|  | *Oxytropis pellita* Bunge | Fabaceae Lindl. | China | Roskov et al. 2006 |
|  | *Oxytropis pseudofrigida* Saposhn. | Fabaceae Lindl. | China | POWO |
|  | *Oxytropis rhynchophysa* Schrenk | Fabaceae Lindl. | Mongolia | POWO |
|  | *Oxytropis sarkandensis* Vassilcz. | Fabaceae Lindl. | China | Roskov et al. 2006 |
|  | *Oxytropis saurica* Saposhn. | Fabaceae Lindl. | China | Roskov et al. 2006 |
|  | *Oxytropis schrenkii* Trautv. | Fabaceae Lindl. | China | Roskov et al. 2006 |
|  | *Oxytropis semenowii* Bunge | Fabaceae Lindl. | China | Roskov et al. 2006 |
|  | *Oxytropis spinifer* Vassilcz. | Fabaceae Lindl. | China | Roskov et al. 2006 |
|  | *Oxytropis talassica* Gontsch. | Fabaceae Lindl. | Kyrgyzstan | POWO |
|  | *Oxytropis ugamensis* Vassilcz. | Fabaceae Lindl. | Uzbekistan | Tojibaev et al. 2020a |
|  | *Oxytropis ugamica* Gontsch. | Fabaceae Lindl. | Tadzhikistan, Uzbekistan | Tojibaev et al. 2020a |
|  | *Gentiana dschungarica* Regel | Gentianaceae Juss. | Russia | POWO |
|  | *Iris alberti* Regel | Iridaceae Juss. | Kyrgyzstan, Uzbekistan | Sennikov et al. 2023 |
|  | *Iris willmottiana* Foster | Iridaceae Juss. | Kyrgyzstan | Lazkov and Sennikov 2017b |
|  | *Dracocephalum karataviense* Pavlov & Roldugin | Lamiaceae Martinov | Uzbekistan | Tojibaev et al. 2020a |
|  | *Hyssopus macranthus* Boriss. | Lamiaceae Martinov | China, Russia | POWO |
|  | *Lagochilus acutilobus* (Ledeb.) Fisch. & C.A.Mey. | Lamiaceae Martinov | Russia, Uzbekistan | POWO |
|  | *Lagochilus bungei* Benth. | Lamiaceae Martinov | China, Mongolia | POWO |
|  | *Lagochilus subhispidus* Knorring | Lamiaceae Martinov | Kyrgyzstan, Uzbekistan | Sennikov and Tojibaev 2021 |
|  | *Nepeta transiliensis* Pojark. | Lamiaceae Martinov | China | Grubov 1970 |
|  | *Phlomoides goloskokovii* Lazkov | Lamiaceae Martinov | Kyrgyzstan | POWO |
|  | *Phlomoides karatavica* (Pavlov) Lazkov & Sennikov | Lamiaceae Martinov | Kyrgyzstan | POWO |
|  | *Phlomoides paniculata* (Regel) Salmaki | Lamiaceae Martinov | Uzbekistan | Hassler (1994 - 2024) |
|  | *Phlomoides sewerzovii* (Herder) Mathiesen | Lamiaceae Martinov | Kyrgyzstan | Lazkov and Sultanova, 2011 |
|  | *Phlomoides zenaidae* (Popov) Adylov, Kamelin & Makhm. | Lamiaceae Martinov | China | POWO |
|  | *Scutellaria flabellulata* Juz. | Lamiaceae Martinov | Kyrgyzstan | Hassler (1994 - 2024) |
|  | *Thymus altaicus* Klokov & Des.-Shost. | Lamiaceae Martinov | China, Mongolia, Russia | POWO |
|  | *Thymus lavrenkoanus* Klokov | Lamiaceae Martinov | Russia | Hassler (1994 - 2024) |
|  | *Thymus narymensis* Serg. | Lamiaceae Martinov | Russia, Mongolia | POWO |
|  | *Thymus petraeus* Serg. | Lamiaceae Martinov | China, Mongolia, Russia | POWO |
|  | *Thymus rasitatus* Klokov | Lamiaceae Martinov | Russia | POWO |
|  | *Gagea azutavica* Kotukhov | Liliaceae Juss. | Uzbekistan | Sennikov and Tojibaev 2021 |
|  | *Gagea michaelis* Golosk. | Liliaceae Juss. | Kyrgyzstan | POWO |
|  | *Gagea pseudominutiflora* Levichev | Liliaceae Juss. | Kyrgyzstan | POWO |
|  | *Gagea ugamica* Pavlov | Liliaceae Juss. | Uzbekistan | Tojibaev et al. 2020a |
|  | *Gagea neopopovii* Golosk. | Liliaceae Juss. | China | Wu et al. 2008 |
|  | *Tulipa borszczowii* Regel | Liliaceae Juss. | Uzbekistan | Tojibaev et al. 2022 |
|  | *Tulipa dasystemonoides* Vved. | Liliaceae Juss. | Kyrgyzstan, Tadzhikistan, Uzbekistan | Tojibaev et al. 2022 |
|  | *Tulipa greigii* Regel | Liliaceae Juss. | Kyrgyzstan, Tadzhikistan, Uzbekistan | POWO, Hassler (1994 - 2024) |
|  | *Tulipa heteropetala* Ledeb. | Liliaceae Juss. | China, Russia | POWO |
|  | *Tulipa ostrowskiana* Regel | Liliaceae Juss. | Kyrgyzstan | POWO |
|  | *Tulipa tarda* Stapf | Liliaceae Juss. | Kyrgyzstan | Hassler (1994 - 2024) |
|  | *Euphrasia drosophylla* Juz. | Orobanchaceae Vent. | Kyrgyzstan | POWO |
|  | *Euphrasia peduncularis* Juz. | Orobanchaceae Vent. | Kyrgyzstan | POWO |
|  | *Pedicularis karatavica* Pavlov | Orobanchaceae Vent. | Kyrgyzstan | POWO |
|  | *Pedicularis songarica* Schrenk ex Fisch. & C.A.Mey. | Orobanchaceae Vent. | China | POWO |
|  | *Corydalis ainae (*Rukšāns ex Lidén) Lazkov & Sennikov | Papaveraceae Juss. | Kyrgyzstan, Uzbekistan | Lazkov and Sennikov, 2017a |
|  | *Corydalis pseudoalpestris* Popov | Papaveraceae Juss. | China | POWO |
|  | *Papaver canescens* Tolm. | Papaveraceae Juss. | China, Mongolia, Russia | POWO |
|  | *Papaver tenellum* Tolm. | Papaveraceae Juss. | Russia | POWO |
|  | *Papaver tianschanicum* Popov | Papaveraceae Juss. | Kyrgyzstan, Russia | POWO |
|  | *Linaria leptoceras* Kuprian. | Plantaginaceae Juss. | Iran | POWO |
|  | *Linaria pedicellata* Kuprian. | Plantaginaceae Juss. | Mongolia | Baasanmunkh et al. 2022; Hassler (1994 - 2024) |
|  | *Veronica arenosa* (Serg.) Boriss. | Plantaginaceae Juss. | China, Mongolia | Wu et al. 2008; Baasanmunkh et al., 2022 |
|  | *Veronica luetkeana* Rupr. | Plantaginaceae Juss. | Kyrgyzstan, Tadzhikistan, Uzbekistan | Hassler (1994 - 2024) |
|  | *Acantholimon tarbagataicum* Gamajun. | Plumbaginaceae Juss. | China | Wu et al. 2008 |
|  | *Acantholimon titovii* Lincz. | Plumbaginaceae Juss. | Kyrgyzstan | POWO |
|  | *Limonium chrysocomum* (Kar. & Kir.) Kuntze | Plumbaginaceae Juss. | China, Mongolia, Russia | POWO |
|  | *Limonium cretaceum*Cherkasova | Plumbaginaceae Juss. | Russia | POWO |
|  | *Limonium leptophyllum* (Schrenk) Kuntze | Plumbaginaceae Juss. | Kyrgyzstan | POWO |
|  | *Agropyron kasteki* Popov | Poaceae Barnhart | Kyrgyzstan | Lazkov and Sultanova 2011 |
|  | *Elymus karakabinicus* Kotukhov | Poaceae Barnhart | Mongolia | POWO |
|  | *Festuca goloskokovii* E.B.Alexeev | Poaceae Barnhart | China | Wu et al. 2008 |
|  | *Leymus aemulans* (Nevski) Tzvelev | Poaceae Barnhart | China, Kyrgyzstan, Turkmenistan, Uzbekistan | POWO |
|  | *Leymus flexilis* (Nevski) Tzvelev | Poaceae Barnhart | Kyrgyzstan | POWO |
|  | *Leymus tianschanicus* (Drobow) Tzvelev | Poaceae Barnhart | China, Iran, Kyrgyzstan, Tadzhikistan, Turkmenistan, Uzbekistan | POWO |
|  | *Stipa × manrakica* Kotukhov | Poaceae Barnhart | Kyrgyzstan | POWO |
|  | *Stipa karataviensis* Roshev. | Poaceae Barnhart | Uzbekistan | Nobis et al. 2020 |
|  | *Stipa macroglossa* subsp. *kazachstanica* (Kotukhov) M.Nobis | Poaceae Barnhart | China, Kyrgyzstan | Nobis et al. 2020 |
|  | *Stipa* *orientalis* var. *azutavica* (Kotukhov) M.Nobis & P.D.Gudkova | Poaceae Barnhart | Mongolia, Pakistan, Russia | Nobis et al. 2020 |
|  | *Stipa sczerbakovii* Kotukhov | Poaceae Barnhart | China, Mongolia, Russia | Nobis et al. 2020 |
|  | *Stipa talassica* Pazij | Poaceae Barnhart | Kyrgyzstan | POWO |
|  | *Atraphaxis canescens* Bunge | Polygonaceae Juss. | China | POWO |
|  | *Atraphaxis decipiens* Jaub. & Spach | Polygonaceae Juss. | China, Russia, Uzbekistan | POWO |
|  | *Calligonum × barsukiense* Soskov | Polygonaceae Juss. | Uzbekistan | Soskov 2011 |
|  | *Calligonum × dissectum* Popov | Polygonaceae Juss. | China | Ma and Xu 2019 |
|  | *Calligonum × spinulosum* Drobow | Polygonaceae Juss. | Uzbekistan | POWO |
|  | *Calligonum crispum* Bunge | Polygonaceae Juss. | China | Ma and Xu 2019 |
|  | *Calligonum triste* Litv. | Polygonaceae Juss. | Turkmenistan, Uzbekistan | POWO |
|  | *Dryopteris mindshelkensis* Pavlov | Polypodiaceae J.Presl & C.Presl | Afghanistan, Albania, Algeria, Bulgaria, France, Great Britain, Greece, Iran, Italy, Romania, Spain, Transcaucasus, Turkey, Yugoslavia | POWO |
|  | *Stuckenia macrocarpa* (Dobrocz.) Tzvelev | Potamogetonaceae Bercht. & J.Presl | Russia | POWO |
|  | *Primula knorringiana* Fed. | Primulaceae Batsch ex Borkh. | Kyrgyzstan | POWO |
|  | *Primula minkwitziae* W.W.Sm. | Primulaceae Batsch ex Borkh. | Kyrgyzstan, Tadzhikistan, Uzbekistan | POWO |
|  | *Delphinium cyananthum* Nevski | Ranunculaceae Juss. | Russia | POWO |
|  | *Delphinium sauricum* Schischk. | Ranunculaceae Juss. | China | Ma and Xu 2019 |
|  | *Ranunculus dilatatus* Ovcz. | Ranunculaceae Juss. | Kyrgyzstan | POWO |
|  | *Ranunculus meinshausenii* Schrenk | Ranunculaceae Juss. | Tadzhikistan | Byalt and Bubyreva 2014 |
|  | *Ranunculus talassicus* Schegol. & A. L. Ebel | Ranunculaceae Juss. | Kyrgyzstan, Uzbekistan | Shchegoleva et al. 2019b |
|  | *Ranunculus transiliensis* Popov ex Gamajun. | Ranunculaceae Juss. | China, Kyrgyzstan | POWO |
|  | *Alchemilla lipschitzii* Lipsch. ex Juz. | Rosaceae Juss. | Kyrgyzstan, Russia | POWO |
|  | *Alchemilla rubens* Lipsch. ex Juz. | Rosaceae Juss. | Russia | POWO |
|  | *Alchemilla sauri* Juz. | Rosaceae Juss. | Russia | Zolotukhin and Chkalov 2019 |
|  | *Alchemilla transiliensis* Juz. | Rosaceae Juss. | Kyrgyzstan | POWO |
|  | *Cotoneaster karatavicus*Pojark. | Rosaceae Juss. | Kyrgyzstan | POWO |
|  | *Cotoneaster oliganthus* Pojark. | Rosaceae Juss. | China, Kyrgyzstan, Tadzhikistan, Uzbekistan | POWO |
|  | *Cotoneaster pseudomultiflorus* Popov | Rosaceae Juss. | China, Uzbekistan | POWO |
|  | *Crataegus almaatensis* Pojark. | Rosaceae Juss. | Kyrgyzstan | POWO |
|  | *Potentilla fedtschenkoana* Siegfr. ex Th.Wolf | Rosaceae Juss. | Kyrgyzstan, Uzbekistan | POWO |
|  | *Potentilla turgaica* Soják | Rosaceae Juss. | Russia | Kurtto et al. 2004 |
|  | *Prunus ledebouriana* (Schltdl.) Y.Y.Yao | Rosaceae Juss. | China | Ma and Xu 2019 |
|  | *Haplophyllum dshungaricum* Rubtzov | Rutaceae Juss. | China | GBIF |
|  | *Thesium minkwitzianum* B.Fedtsch. | Santalaceae R.Br. | Kyrgyzstan | Lazkov and Sultanova 2011 |
|  | *Tamarix kasakhorum* Gorschk. | Tamaricaceae Link | Mongolia | Baasanmunkh et al. 2022 |
|  | *Diarthron tarbagataicum* (Pobed.) Kit Tan | Thymelaeaceae Juss. | China | – |
|  | *Diarthron tianschanicum* (Pobed.) Kit Tan | Thymelaeaceae Juss | China, Kyrgyzstan | POWO |
|  | *Zygophyllum cuspidatum* Boriss. | Zygophyllaceae R.Br. | China | GBIF |
|  | *Zygophyllum fabagoides* Popov | Zygophyllaceae R.Br. | China | POWO |
|  | *Zygophyllum iliense* Popov | Zygophyllaceae R.Br. | China | POWO |
|  | *Zygophyllum kegense* Boriss. | Zygophyllaceae R.Br. | Kyrgyzstan | POWO |
