# Supplementary material for: ﻿Revised checklist of endemic vascular plants of Kazakhstan
Source: PhytoKeys. 2024 Feb 28;238:241–79. doi: 10.3897/phytokeys.238.114475 (PMC10918586; doi:10.3897/phytokeys.238.114475)
Supplement: Supplementary material 2 — Former endemics of Kazakhstan that are now reclassified as synonyms for species exhibiting broader geographical distributions [file phytokeys-238-241_article-114475__-s002.docx]

**Supplementary data 2**

Former endemics of Kazakhstan that are now reclassified as synonyms for species exhibiting broader geographical distributions

| № | **Species name** | **Family** | **Synonyms** | **Reference** |
| --- | --- | --- | --- | --- |
|  | *Anabasis ramosissima* Minkw. | Amaranthaceae Juss. | synonym of *Anabasis salsa* (Ledeb.) Benth. ex Volkens | POWO |
|  | *Allium aemulans* Pavlov | Amaryllidaceae J.St.-Hil. | synonym of *Allium caesium* Schrenk | POWO |
|  | *Allium albertii* Regel | Amaryllidaceae J.St.-Hil. | synonym of *Allium pallasii* Murray | POWO |
|  | *Allium amblyophyllum* Kar. & Kir. | Amaryllidaceae J.St.-Hil. | synonym of *Allium platyspathum* subsp*. amblyophyllum* (Kar. & Kir.) N.Friesen | POWO |
|  | *Allium caricoides* Regel | Amaryllidaceae J.St.-Hil. | synonym of *Allium kokanicum* Regel | POWO |
|  | *Allium kurdaicum* Bajtenov | Amaryllidaceae J.St.-Hil. | synonym of *Allium sewerzowii* Regel | POWO |
|  | *Allium renardii* Regel | Amaryllidaceae J.St.-Hil. | synonym of *Allium caesium* Schrenk | POWO |
|  | *Allium ubinicum* Kotukhov | Amaryllidaceae J.St.-Hil | synonym of *Allium schoenoprasum* L. | POWO |
|  | *Aulacospermum alatum* (Korovin) Korovin | Apiaceae Lindl. | synonym of *Lomatocarpa alata* (Korovin) Pimenov & Sennikov | POWO |
|  | *Aulacospermum rupestre* Popov | Apiaceae Lindl. | synonym of *Aulacospermum* *simplex* Rupr. | Hassler (1994 - 2024) |
|  | *Cryptodiscus arenarius* Schischk. | Apiaceae Lindl. | synonym of *Prangos cachroides* (Schrenk) Pimenov & V.N.Tikhom. | POWO |
|  | *Elaeopleurum monococcum* Korovin | Apiaceae Lindl. | synonym of *Seseli hippomarathrum* subsp. *hebecarpum* Drude | POWO |
|  | *Ferula arida* (Korovin) Korovin | Apiaceae Lindl. | synonym of *Ferula songarica* Pall. ex Willd. | POWO |
|  | *Ferula eremophila* Korovin | Apiaceae Lindl. | synonym of *Ferula karatavica* Regel & Schmalh. | POWO |
|  | *Libanotis petrophila* Korovin | Apiaceae Lindl. | synonym of *Phlojodicarpus villosus* (Turcz. ex Fisch. & C.A.Mey.) Turcz. ex Ledeb. | POWO |
|  | *Oedibasis karatavica* Korovin | Apiaceae Lindl. | synonym of *Oedibasis platycarpa* (Lipsky) Koso-Pol. | POWO |
|  | *Seseli squarrosum* Schischk. | Apiaceae Lindl. | synonym of *Seseli valentinae* Popov | POWO |
|  | *Talassia renardii* (Regel & Schmalh.) Korovin | Apiaceae Lindl. | synonym of *Ferula renardii* (Regel & Schmalh.) Pimenov | POWO |
|  | *Vincetoxicum mugodsharicum* Pobed. | Apocynaceae Juss. | synonym of *Vincetoxicum fuscatum* subsp. *fuscatum* | POWO |
|  | *Artemisia albicerata* Krasch. | Asteraceae Bercht. & J.Presl | synonym of *Artemisia arenaria* DC. | POWO |
|  | *Artemisia glabella* Kar. & Kir. | Asteraceae Bercht. & J.Presl | synonym of *Artemisia obtusiloba* var. *glabra* | POWO |
|  | *Centaurea bipinnatifida* (Trautv.) Gamajun. | Asteraceae Bercht. & J.Presl | synonym of *Rhaponticoides ruthenica* (Lam.) M.V.Agab. & Greuter. | POWO |
|  | *Jurinea altaica* Iljin | Asteraceae Bercht. & J.Presl | synonym of *Jurinea polyclonos* (L.) DC. | Kupriyanov 2018 |
|  | *Jurinea spiridonovii* Iljin | Asteraceae Bercht. & J.Presl | synonym of *Jurinea multiflora* (L.) B.Fedtsch. | Kupriyanov 2018 |
|  | *Lepidolopha filifolia* Pavlov | Asteraceae Bercht. & J.Presl | synonym of *Lepidolopha komarowii* C.Winkl. | POWO |
|  | *Scorzonera rubroviolacea* Godwinski | Asteraceae Bercht. & J.Presl | synonym of *Scorzonera sericeolanata* (Bunge) Krasch. & Lipsch. | POWO |
|  | *Serratula angulata* Kar. & Kir. | Asteraceae Bercht. & J.Presl | synonym of *Klasea dissecta* (Ledeb.) L.Martins | POWO |
|  | *Serratula dshungarica* Iljin | Asteraceae Bercht. & J.Presl | synonym of *Klasea marginata* (Tausch) Kitag. | POWO |
|  | *Taraxacum rubtzovii* Schischk. | Asteraceae Bercht. & J.Presl | synonym of *Taraxacum songoricum* Schischk. | POWO |
|  | *Berberis bykoviana* Pavlov | Berberidaceae Juss. | synonym of *Berberis heteropoda* Schrenk | Hassler (1994 - 2024) |
|  | [*Berberis iliensis*Popov](https://powo.science.kew.org/taxon/urn:lsid:ipni.org:names:106771-1) | Berberidaceae Juss. | synonym of *Berberis integerrima* Bunge | POWO |
|  | *Betula crassijulis* Musceg. | Betulaceae Gray | synonym of *Betula tianschanica* Rupr. | POWO |
|  | *Betula kirghisorum* Sawicz | Betulaceae Gray | synonym of *Betula tianschanica* Rupr. | POWO |
|  | *Betula talassica* Poljakov | Betulaceae Gray | synonym of *Betula pendula* subsp.*pendula* | POWO |
|  | *Craniospermum echioides* (Schrenk) Bunge | Boraginaceae Juss. | synonym of *Craniospermum subvillosum* Lehm. | POWO |
|  | *Lepechiniella balchaschensis* Popov | Boraginaceae Juss. | synonym of *Lepechiniella lasiocarpa* W.T.Wang | POWO |
|  | *Lindelofia angustifolia* (Schrenk) Brand | Boraginaceae Juss. | synonym of *Lindelofia stylosa* subsp. *stylosa* | POWO |
|  | *Mertensia popovii* N.I.Rubtzov | Boraginaceae Juss. | synonym of *Mertensia meyeriana* J.F.Macbr. | POWO |
|  | *Paracaryum petrophilum* Pavlov ex Golosk. | Boraginaceae Juss. | synonym of *Microparacaryum intermedium* subsp. *intermedium* | POWO |
|  | *Draba bajtenovii* Vesselova | Brassicaceae Burnett | synonym of *Draba fladnizensis* Wulfen | German 2022 |
|  | *Isatis frutescens* Kar. & Kir. | Brassicaceae Burnett | synonym of *Isatis costata* C.A. Mey. | German 2006b; Kiefer et al. 2014 |
|  | *Isatis maxima* Pavlov | Brassicaceae Burnett | synonym of *Isatis emarginata* Kar. & Kir. | Kiefer et al. 2014; Veselova 2016 |
|  | *Lepidium deserti* Pavlov | Brassicaceae Burnett | synonym of *Lepidium obtusum* Basiner | POWO; German 2014 |
|  | *Lepidium eremophilum* Schrenk | Brassicaceae Burnett | synonym of *Lepidium songaricum* Schrenk ex Fisch. & C.A.Mey. | Vinogradova 1974 |
|  | *Lepidium rubtzovii* Vassilcz. | Brassicaceae Burnett | synonym of *Lepidium lacerum* subsp*. rubtzovii* (Vassiclz.) D.A. German | POWO; German, 2014 |
|  | *Megacarpaea iliensis* Golosk. & Vassilcz. | Brassicaceae Burnett | synonym of *Megacarpaea megalocarpa* (Fisch. ex DC.) Schischk. ex B.Fedtsch. | Kiefer et al. 2014 |
|  | *Megacarpaea mugodzharica* Golosk. & Vassilcz. | Brassicaceae Burnett | synonym of *Megacarpaea megalocarpa* (Fisch. ex DC.) Schischk. ex B.Fedtsch. | Kiefer et al. 2014 |
|  | *Sterigmostemum schmakovii* Kamelin & D.A.German | Brassicaceae Burnett | synonym of *Sterigmostemum fuhaiense* H.L. Yang | German and Al-Shehbaz 2017 |
|  | *Torularia karatavica* Myrz. & Bajtenov | Brassicaceae Burnett | synonym of *Strigosella scorpioides* (Bunge) Botsch. | POWO; German 2016 |
|  | *Lonicera karataviensis Pavlov* | Caprifoliaceae Juss. | synonym of *Lonicera tatarica L.* | POWO |
|  | *Lonicera popovii* Golosk. | Caprifoliaceae Juss. | synonym of *Lonicera humilis* Kar. & Kir. | POWO |
|  | *Melandrium sordidum* (Kar. & Kir.) Rohrb. | Caryophyllaceae Juss. | synonym of *Silene karekirii* Bocquet | POWO |
|  | *Silene balchaschensis* Schischk. | Caryophyllaceae Juss. | synonym of *Silene gavrilovii* (Krasn.) Popov | POWO |
|  | *Silene holopetala* Bunge | Caryophyllaceae Juss. | synonym of *Silene sibirica* (L.) Pers. | POWO |
|  | *Silene heptapotamica* Schischk. | Caryophyllaceae Juss. | synonym of *Silene claviformis* Litv. | POWO |
|  | *Silene karkaralensis* Dmitrieva & Popov | Caryophyllaceae Juss. | synonym of *Silene stylosa* Bunge | POWO; Belkin 2009 |
|  | *Silene kirgisensis* Bajtenov & Nelina | Caryophyllaceae Juss. | synonym of *Silene longicarpophora* (Kom.) Bocquet | Sennikov and Tojibaev 2021 |
|  | *Stellaria alatavica* Popov | Caryophyllaceae Juss. | synonym of *Stellaria brachypetala* Bunge | POWO |
|  | *Rhodiola viridula* Boriss. | Crassulaceae J.St.-Hil. | synonym of *Rhodiola heterodonta* (Hook.f. & Thomson) Boriss. | POWO |
|  | *Juniperus talassica* Lipsky | Cupressaceae Gray | synonym of *Juniperus semiglobosa* Regel | POWO |
|  | *Kobresia smirnovii* N.A.Ivanova | Cyperaceae Juss. | synonym of *Carex borealipolaris* S.R.Zhang | POWO |
|  | *Euphorbia prokhanovii* Popov | Euphorbiaceae Juss. | synonym of *Euphorbia thomsoniana* Boiss. | POWO |
|  | *Euphorbia subamplexicaulis* Kar. & Kir. | Euphorbiaceae Juss. | synonym of *Euphorbia buchtormensis* C.A.Mey. | Hassler (1994 - 2024) |
|  | *Astragalus anrachaicus*Golosk. | Fabaceae Lindl. | synonym of *Astragalus larvatus* Sumnev. | POWO |
|  | *Astragalus chaeturus* Popov | Fabaceae Lindl. | synonym of *Astragalus stenocystis* Bunge | POWO |
|  | *Astragalus czuiliensis* Golosk. | Fabaceae Lindl. | synonym of *Astragalus globiceps* Bunge | POWO |
|  | *Astragalus poliotes* Bunge | Fabaceae Lindl. | synonym of *Astragalus testiculatus* Pall. | POWO |
|  | *Astragalus transiliensis* Gontsch. | Fabaceae Lindl. | synonym of *Astragalus ellipsoideus* Ledeb. | POWO |
|  | *Astragalus trautvetteri* Bunge | Fabaceae Lindl. | synonym of *Astragalus schanginianus* Pall. | POWO |
|  | *Hedysarum dshambulicum* Pavlov | Fabaceae Lindl. | synonym of *Hedysarum plumosum* Boiss. & Hausskn. | POWO |
|  | *Hedysarum kandyktassicum* Bajtenov | Fabaceae Lindl. | synonym of *Hedysarum iliense* B.Fedtsch. ex Popov | POWO |
|  | *Hedysarum kasteki* Bajtenov. | Fabaceae Lindl. | synonym of *Hedysarum plumosum* Boiss. & Hausskn. | Hassler (1994 - 2024) |
|  | *Medicago × komarovii* Vassilcz. | Fabaceae Lindl. | synonym of *Medicago × varia* Martyn | POWO |
|  | *Medicago × subdicycla* (Trautv.) Vassilcz. | Fabaceae Lindl. | synonym of *Medicago × varia* Martyn | POWO |
|  | *Medicago × trautvetteri* Sumnev. | Fabaceae Lindl. | synonym of *Medicago × varia* Martyn | POWO |
|  | *Meristotropis erythrocarpa* Vassilcz. | Fabaceae Lindl. | synonym of *Glycyrrhiza triphylla* Fisch. & C.A.Mey. | POWO |
|  | *Oxytropis goloskokovii* Bajtenov | Fabaceae Lindl. | synonym of *Oxytropis gorbunovii* Boriss. | POWO |
|  | *Oxytropis robusta* Popov | Fabaceae Lindl. | synonym of *Oxytropis macrocarpa* Kar. & Kir. | POWO |
|  | *Oxytropis tujaksuensis* Bajtenov | Fabaceae Lindl. | synonym of *Oxytropis sewerzowii* Bunge | POWO |
|  | *Ribes turbinatum Pojark.* | Grossulariaceae DC. | synonym of *Ribes nigrum* L. | Grubov 2007 |
|  | *Iris almaatensis* Pavlov | Iridaceae Juss. | synonym of *Iris kuschakewiczii* B.Fedtsch. | Sennikov et al. 2023 |
|  | *Iris coerulea* B. Fedtsch. | Iridaceae Juss. | synonym of *Iris albomarginata* R.C.Foster | POWO |
|  | *Iris haematophyla* Fisch. ex Link | Iridaceae Juss. | synonym of *Iris lactea* f*. biglumis* (Vahl) Kitag. | POWO |
|  | *Scutellaria catharinae* Juz. | Lamiaceae Martinov | synonym of *Scutellaria sieversii* Bunge | Hassler (1994 - 2024) |
|  | *Scutellaria irregularis* Juz. | Lamiaceae Martinov | synonym of *Scutellaria supina* L. | POWO |
|  | *Scutellaria soongorica* Juz. | Lamiaceae Martinov | synonym of *Scutellaria sieversii* Bunge | POWO |
|  | *Thymus irtyschensis* Klokov | Lamiaceae Martinov | synonym of *Thymus mongolicus* (Ronniger) Ronniger | POWO |
|  | *Thymus kasakstanicus* Klokov & Des.-Shost. | Lamiaceae Martinov | synonym of *Thymus kirgisorum* Dubj. | POWO |
|  | *Gagea obvoluta* Pavlov | Liliaceae Juss. | synonym of *Gagea afghanica* A.Terracc. | POWO |
|  | *Tulipa behmiana* Regel | Liliaceae Juss. | synonym of *Tulipa lehmanniana* Merckl. | POWO |
|  | *Trapa kasachstanica* V.N.Vassil. | Lythraceae J.St.-Hil. | synonym of *Trapa natans* var. *natans* | POWO |
|  | *Trapa saissanica* (Flerow) V.N.Vassil. | Lythraceae J.St.-Hil. | synonym of *Trapa natans* var*. natans* | POWO |
|  | *Orobanche brevidens* Novopokr. | Orobanchaceae Vent. | synonym of *Orobanche cernua* Loefl. | POWO |
|  | *Orobanche karatavica* Pavlov | Orobanchaceae Vent. | synonym of *Phelipanche pallens* (Bunge ex Ledeb.) Soják | GBIF |
|  | *Pedicularis jugentassica* Semiotr. | Orobanchaceae Vent. | synonym of *Pedicularis dolichorrhiza* Schrenk | POWO |
|  | *Linaria bektauatensis* Semiotr. | Plantaginaceae Juss. | synonym of *Linaria altaica* Fisch. | POWO |
|  | *Linaria brachyceras* (Bunge) Kuprian. | Plantaginaceae Juss. | synonym of *Linaria altaica* Fisch. | POWO |
|  | *Linaria dmitrievae* Semiotr. | Plantaginaceae Juss. | synonym of *Linaria altaica* Fisch. | POWO |
|  | *Linaria dolichocarpa* Klokov | Plantaginaceae Juss. | synonym of *Linaria odora* (M.Bieb.) Fisch. | POWO |
|  | *Linaria ramosa* (Kar. & Kir.) Kuprian. | Plantaginaceae Juss. | synonym of *Linaria bungei* Kuprian. | POWO |
|  | *Veronica chantavica* Pavlov | Plantaginaceae Juss. | synonym of *Veronica biloba* Schreb. ex L. | POWO |
|  | *Agropyron atbassaricum* Golosk. | Poaceae Barnhart | synonym of *Elymus violaceus* (Hornem.) J.Feilberg | POWO |
|  | *Agropyron dshungaricum* (Nevski) Nevski | Poaceae Barnhart | synonym of  *Pseudoroegneria geniculata* (Trin.) Á.Löve | POWO |
|  | *Agropyron karataviense* Pavlov | Poaceae Barnhart | synonym of  *Agropyron cristatum* (L.) Gaertn. | POWO |
|  | *Agropyron karkaralense* Roshev. | Poaceae Barnhart | synonym of  *Elymus uralensis* (Nevski) Tzvelev | POWO |
|  | *Agropyron transiliense* Popov | Poaceae Barnhart | synonym of *Elymus mutabilis* (Drobow) Tzvelev | POWO |
|  | *Agrostis buchtarmensis* Kotukhov | Poaceae Barnhart | synonym of *Agrostis vinealis* Schreb. | POWO |
|  | *Bromus pskemensis* Pavlov | Poaceae Barnhart | synonym of *Bromus inermis* Leyss. | POWO |
|  | *Calamagrostis gigantea* Roschev. | Poaceae Barnhart | synonym of *Calamagrostis macrolepis* Litv. | POWO |
|  | *Calamagrostis karataviensis* P.A.Smirn. | Poaceae Barnhart | synonym of *Calamagrostis macrolepis* Litv. | Hassler (1994 - 2024) |
|  | *Cinna karataviensis* Pavlov | Poaceae Barnhart | synonym of *Agrostis giganthea* subsp. *gigantea* | POWO |
|  | *Elymus besczetnovae* Kotukhov | Poaceae Barnhart | synonym of *Elymus macrourus* (Turcz. ex Steud.) Tzvelev | POWO |
|  | *Elymus buchtarmensis* Kotukhov | Poaceae Barnhart | synonym of *Elymus fedtschenkoi* Tzvelev | POWO |
|  | *Elymus goloskokovii* Kotukhov | Poaceae Barnhart | synonym of *Elymus fibrosus* (Schrenk) Tzvelev | POWO |
|  | *Elymus karataviensis* Roshev. | Poaceae Barnhart | synonym of *Leymus alaicus* (Korsh.) Tzvelev | POWO |
|  | *Elymus kuznetzovii* Pavlov | Poaceae Barnhart | synonym of *Leymus karelinii* (Turcz.) Tzvelev | POWO |
|  | *Elymus lineicus* Kotukhov | Poaceae Barnhart | synonym of *Elymus mutabilis* (Drobow) Tzvelev | POWO |
|  | *Elymus longispicatus* Kotukhov | Poaceae Barnhart | synonym of *Elymus caninus (*L.) L. | POWO |
|  | *Elymus marmoreus* Kotukhov | Poaceae Barnhart | synonym of *Elymus mutabilis* (Drobow) Tzvelev | POWO |
|  | *Elymus occidentalialtaicus* Kotukhov | Poaceae Barnhart | synonym of *Elymus mutabilis* (Drobow) Tzvelev | POWO |
|  | *Elymus petraeus* (Nevski) Pavlov | Poaceae Barnhart | synonym of *Leymus alaicus* (Korsh.) Tzvelev | POWO |
|  | *Elymus sarymsactensis* Kotukhov | Poaceae Barnhart | synonym of *Elymus gmelinii* (Trin.) Tzvelev | POWO |
|  | *Elymus sauricus* Kotukhov | Poaceae Barnhart | synonym of *Elymus abolinii* (Drobow) Tzvelev | POWO |
|  | *Elymus tarbagataicus* Kotukhov | Poaceae Barnhart | synonym of *Elymus gmelinii* (Trin.) Tzvelev | POWO |
|  | *Elymus tzvelevii* Kotukhov | Poaceae Barnhart | synonym of *Campeiostachys schrenkiana* (Fisch. & C.A.Mey. ex Schrenk) Drobow | POWO |
|  | *Elymus ubinicus* Kotukhov | Poaceae Barnhart | synonym of *Elymus mutabilis* (Drobow) Tzvelev | POWO |
|  | *Elytrigia czindogatuica* Kotukhov | Poaceae Barnhart | synonym of *Elymus gmelinii* (Trin.) Tzvelev | POWO |
|  | *Eremopoa glareosa* Gamajun. | Poaceae Barnhart | synonym of *Poa diaphora* var*. songarica* (Schrenk) Soreng, Cabi & L.J.Gillespie | POWO |
|  | *Festuca erectiflora* Pavlov | Poaceae Barnhart | synonym of *Festuca amblyodes* V.I.Krecz. & Bobrov | POWO |
|  | *Koeleria transiliensis* Reverd. ex Gamajun. | Poaceae Barnhart | synonym of *Koeleria macrantha* subsp. *macrantha* | POWO |
|  | *Phleum roshevitzii* Pavlov | Poaceae Barnhart | synonym of *Phleum pratense L*. | POWO |
|  | *Piptatherum karataviense* Roshev. | Poaceae Barnhart | synonym of *Piptatherum holciforme* subsp*. holciforme* | POWO |
|  | *Poa insignis* Litv. | Poaceae Barnhart | synonym of *Poa sibirica* Roshev. | POWO |
|  | *Poa korshunensis* Golosk. | Poaceae Barnhart | synonym of *Poa urssulensis* Trin. | POWO |
|  | *Poa kungeica* Golosk. | Poaceae Barnhart | synonym of *Poa lipskyi* Roshev. | POWO |
|  | *Poa roshevitzii* Golosk. | Poaceae Barnhart | synonym of *Poa alberti* subsp. *alberti* | POWO |
|  | *Stipa akseirica* Kotukhov | Poaceae Barnhart | synonym of *Stipa sareptana* A.K.Becker | POWO |
|  | *Stipa krascheninnikowii* Roshev | Poaceae Barnhart | synonym of *Stipa ucrainica* P.A. Smirn. | POWO |
|  | *Stipa pavlovii* Kotukhov | Poaceae Barnhart | synonym of *Stipa sczerbakovii* Kotukhov | POWO, Nobis et al. 2020 |
|  | *Stipa saikanica* Kotukhov | Poaceae Barnhart | synonym of *Stipa lessingiana* Trin. & Rupr. | POWO, Nobis et al. 2020 |
|  | *Stipa turgaica* Roshev. | Poaceae Barnhart | synonym of *Stipa arabica* Trin. & Rupr. | POWO |
|  | *Calligonum × androsowii* Litv. | Polygonaceae Juss. | synonym of *Calligonum × dubianskyi* Litv. | POWO |
|  | *Calligonum × erinaceum* I.G.Borshch. | Polygonaceae Juss. | synonym of *Calligonum × densum* I.G.Borshch. | POWO |
|  | *Calligonum aculeatum* (Litv.) Mattei | Polygonaceae Juss. | synonym of *Calligonum aphyllum* (Pall.) Gürke | POWO |
|  | *Calligonum babakianum* Godw. | Polygonaceae Juss. | synonym of *Calligonum rubicundum* Bunge | Soskov 2011 |
|  | *Calligonum bykovii* Godw. | Polygonaceae Juss. | synonym of *Calligonum junceum* (Fisch. et C.A.Mey.) Litv. | Soskov 2011 |
|  | *Calligonum coriaceum* Pavlov | Polygonaceae Juss. | synonym of *Calligonum rubicundum* Bunge | POWO |
|  | *Calligonum crispatum* (Litv.) Mattei | Polygonaceae Juss. | synonym of *Calligonum aphyllum* (Pall.) Gürke | POWO |
|  | *Calligonum flavidum* Bunge | Polygonaceae Juss. | synonym of *Calligonum rubicundum* Bunge | POWO |
|  | *Calligonum humile* Litv. | Polygonaceae Juss. | synonym of *Calligonum aphyllum* (Pall.) Gürke | POWO |
|  | *Calligonum involutum* Pavlov | Polygonaceae Juss. | synonym of *Calligonum rubicundum* Bunge | POWO |
|  | *Calligonum kzyl-kumi* Pavlov | Polygonaceae Juss. | synonym of *Calligonum setosum* (Litv.) Litv. | POWO |
|  | *Calligonum lamellatum* (Litv.) Mattei | Polygonaceae Juss. | synonym of *Calligonum aphyllum* (Pall.) Gürke | POWO |
|  | *Calligonum palibinii* Mattei | Polygonaceae Juss. | synonym of *Calligonum aphyllum* (Pall.) Gürke | POWO |
|  | *Calligonum patens* Litv. | Polygonaceae Juss. | synonym of *Calligonum leucocladum* subsp. *leucocladum* | POWO |
|  | *Calligonum plicatum* Pavlov | Polygonaceae Juss. | synonym of *Calligonum leucocladum* subsp. *leucocladum* | POWO |
|  | *Calligonum pseudohumile* Drobow | Polygonaceae Juss. | synonym of *Calligonum aphyllum* (Pall.) Gürke | POWO |
|  | *Calligonum russanovi*i Pavlov | Polygonaceae Juss. | synonym of *Calligonum rubicundum* Bunge | POWO |
|  | *Calligonum tortile* Drobow | Polygonaceae Juss. | synonym of *Calligonum aphyllum* (Pall.) Gürke | POWO |
|  | *Calligonum ustjurtense* Drobow | Polygonaceae Juss. | synonym of *Calligonum aphyllum* (Pall.) Gürke | POWO |
|  | *Aconitum alatavicum* Vorosch. | Ranunculaceae Juss. | synonym of *Aconitum soongaricum* (Regel) Stapf | POWO |
|  | *Anemone almaatensis* Juz. | Ranunculaceae Juss. | synonym of *Anemone biflora* var. *gortschakowii* (Kar. & Kir.) Sinno | POWO |
|  | *Atragene tianschanica* Pavlov | Ranunculaceae Juss. | synonym of *Clematis alpina* subsp*. sibirica* (L.) Kuntze | POWO |
|  | *Delphinium karataviense* Pavlov | Ranunculaceae Juss. | synonym of *Delphinium albomarginatum* Simonova | POWO |
|  | *Thalictrum saissanicus* Kotukhov | Ranunculaceae Juss. | synonym of *Thalictrum minus* subsp. *minus* | POWO |
|  | *Rosa pavlovii* Chrshan. | Rosaceae Juss. | synonym of *Rosa majalis* Herrm. | POWO |
|  | *Sanguisorba riparia* Juz. | Rosaceae Juss. | synonym of *Sanguisorba officinalis* L. | Sennikov and Tojibaev 2021; Wu et al. 2008 |
|  | *Populus berkarensis* Poljakov | Salicaceae Mirb. | synonym of *Populus alba* L. | POWO |
|  | *Parietaria jaxartica* Pavlov | Urticaceae Juss. | synonym of *Parietaria judaica* subsp*. judaica* | POWO |
